# Supplementary material for: Aetiology of lobar pneumonia determined by multiplex molecular analyses of lung and pleural aspirate specimens in the Gambia: findings from population-based pneumonia surveillance
Source: BMJ Open. 2022 Mar 9;12(3):e056706. doi: 10.1136/bmjopen-2021-056706 (PMC8915295; doi:10.1136/bmjopen-2021-056706)
Supplement: Supplementary data [file bmjopen-2021-056706supp001.pdf]

Aetiology of Lobar Pneumonia Determined by Multiplex Molecular Analyses of Lung and Pleural Aspirate Samples in The Gambia: Findings from Population-based Pneumonia Surveillance

Methods for multiplex PCR assay

The laboratory, staff, and methods used to analyse the specimens were the same to those used in the PERCH study which was also conducted in The Gambia at a similar time. Total nucleic acid was extracted from a 200µl aliquot of lung and pleural aspirates (easyMAG, bioMériux, France) with an internal control. Extracts were subjected to quantitative multiplex PCR (Fast-track Diagnostics Resp-33 kit, Sliema, Malta) for a panel of 33 respiratory bacteria, fungi, and viruses with internal positive and negative control. The assay was structured in eight component multiplex sub-assays with three or four targets run on one plate. We used a Bio-Rad CFX96 thermocycler with programming as recommended by the manufacturer. Standard PCR curves were derived from plasmid standards during the testing to calculate pathogen load from cycle threshold values. The multiplex PCR included the following targets:

- *S. pneumoniae* (lytA),
- *Haemophilus influenze* sp. (ompP6),
- *H. influenzae* type b (bexA),
- *S. aureus* (shkv),
- *Chlamydia pneumoniae* (RNApbc),
- *Moraxella catarrhalis* (copB),
- *Klebsiella pneumoniae* (khe),
- *Legionella* sp. (16SrRNA),
- *Pneumocystis jirovecii* (mtlsurRNA),
- *Bordetella pertussis* (is481),
- *Salmonella* sp. (ttrB),
- Influenza A (pos1), B (seg8ns1nep) and C (mtx),
- Cytomegalovirus (us7&8),
- Parainfluenza virus 1 (hnmRNA), 2 (hnmRNA), 3 (hnmRNA) and 4 (fus),
- Rhinovirus (utr),
- Coronaviruses NL63 (ncpn), 229E (ncpn), OC43 (ncpn) and HKU1 (ncpn),
- Respiratory syncytial virus A (nucap) and B (numRNA),
- Metapneumovirus A (fuglF) and B (fuglF),
- Adenovirus (hex),
- Bocavirus (np1),
- Enterovirus (dom4&5),
- Parechovirus (utr),
- *Mycoplasma pneumoniae* (adP1),

Data were not used for *K. pneumoniae* and Legionella spp. Interpretation for some targets required combinations of results: if rhinovirus only was detected then the specimen was deemed rhinovirus positive, whereas if rhinovirus and enterovirus were detected then the specimen was deemed enterovirus positive; if *H. influenzae* type b and *H. influenzae* were detected the specimen was deemed positive for *H. influenzae* type b, whereas if *H. influenzae* only was detected the specimen was deemed positive for *H. influenzae* non-type b.

Clinical characteristics of patients

Table 1 in the manuscript describes the characteristics of the patients in three categories: no lobar consolidation, lobar consolidation and no lung/pleural aspirate, and lobar consolidation and lung/pleural aspirate. Compared to patients without lobar pneumonia, those with lobar pneumonia had greater respiratory rate (p<0.0001), lower oxygen saturation (p=0.034), and less wheeze (p<0.0001), whereas heart rate (p=0.59), temperature (p=0.73), prostration (p=0.25), weight-for-height z-score <-3 in young children (p=0.28) and severe underweight in older children and adults (p=0.36) were not significantly different. Respiratory rate (p=0.50), heart rate (p=0.20), temperature (p=0.12), prostration (p=0.20), weight-for-height z-score <-3 in young children (p=0.62), and severe underweight in older children and adults (p=0.86) were not significantly different in patients with lobar pneumonia who did or did not have a lung aspirate, although wheeze was more frequent in patients without lung aspirate (76/562 versus 11/181, p=0.007) and oxygen saturation was greater (p=0.017). Bacteremia was more likely in patients who had a lung aspirate (31/178, 17%) compared to those without a lung aspirate, irrespective of whether lobar pneumonia was present on chest radiograph (113/2119, 5%). Ninety-six patients died (3.8%) with similar proportions in the three clinical categories.

Quantification of pathogen load

The greatest pathogen load in lung specimens was associated with *S. pneumoniae* (median 5.34 [IQR 3.73, 6.24] log_{10} copies/ml), *H. influenzae* non-type b (median 6.07 [IQR 5.32, 6.86] log_{10} copies/ml) and parainfluenza virus (PIV) 1 (median 6.46 [IQR 4.74, 10.93] log_{10} copies/ml) positive specimens (Supplementary Table 1). Low pathogen load was associated with *S. aureus* (median 2.15 [IQR 1.68, 4.14] log_{10} copies/ml), bocavirus (median 2.77 [IQR 2.19, 3.40] log_{10} copies/ml]), and cytomegalovirus (2.57 [IQR 2.38, 3.71] log_{10} copies/ml) positive specimens.

Supplementary table 1. Organism-specific quantification of pathogen load in 156 lung and 4 pleural aspirate specimens

Organism	Quantification of organism (median[IQR]; min, max); log_{10} copies per ml
**Bacteria**	
Streptococcus pneumoniae (n=68)	5.34 (3.73 – 6.24); 1.44, 9.58
Staphylococcus aureus (n=26)	2.15 (1.68 – 4.14); 1.43, 8.49
Haemophilus influenzae type b (n=11)	4.18 (2.26 – 6.30); 1.56, 9.11
Moraxella catarrhalis (n=8)	4.40 (3.71 – 5.50); 2.63, 6.30
Salmonella species (n=8)	3.01 (1.74 – 5.29); 0.86, 9.07
Haemophilus influenzae non-type b (n=6)	6.07 (5.32 – 6.86); 4.88, 8.21
Bordetella pertussis (n=4)	undef (undef); 0.30, 4.32
Chlamydia pneumonia (n=3)	3.60 (undef); 2.13, 4.73
**Viruses**	
Bocavirus (n=11)	2.77 (2.19 – 3.40); 1.53, 4.76
Parainfluenza 1 (n=8)	6.46 (4.74 – 10.93); 4.32, 12.50
Influenza C (n=7)	4.47 (4.21 – 5.64); 3.72, 6.85
Cytomegalovirus (n=6)	2.57 (2.38 – 3.71); 1.45, 5.89
Coronavirus HKU1 (n=4)	3.93 (undef); 3.77, 4.46
Coronavirus 43 (n=4)	4.77 (undef); 4.25, 5.37
Respiratory syncytial virus (n=3)	6.59 (undef); 5.07, 7.07
**Fungi**	
Pneumocystis jirovecii (n=9)	2.82 (2.52 – 3.37); 2.14, 7.42

Note: organisms listed were detected in three or more of 160 specimens. *B. pertussis* PCR Ct values were too great to allow quantification for three of seven specimens. The results of pathogen quantification in lobar pneumonia are subject to variation in the small volumes of specimen obtained and its dilution in 1ml of sterile saline.

Mackenzie GA, *et al. BMJ Open* 2022; 12:e056706. doi: 10.1136/bmjopen-2021-056706

Effectiveness of PCV to prevent pneumococcal pneumonia

Supplementary table 2. Association of pneumococcal pneumonia with PCV vaccination status

**Pneumonia aetiology by culture of blood or lung/pleural aspirate**	**Number of PCV doses (PCV7 or PCV13)**		**Total N**	**Odds ratio (95% CI)**
	≥2 doses	0 doses		
Age 2-11 months	N=540	N=184		
Culture pneumococcal	5	5	10	
Culture non-pneumococcal	535	179	714	0.33 (0.08, 1.47)
Proportion culture pneumococcal	0.009	0.027	724	
Age 12-23 months	N=515	N=81		
Culture pneumococcal	15	2	17	
Culture non-pneumococcal	500	79	560	1.19 (0.27, 10.9)
Proportion culture pneumococcal	0.029	0.025	577	
Age 2-4 years	N=230	N=218		
Culture pneumococcal	9	15	24	
Culture non-pneumococcal	221	203	424	0.55 (0.21, 1.38)
Proportion culture pneumococcal	0.039	0.069	448	
Combined age strata 2-59 months, [a]M-H age-stratified odds ratio = 0.57 (0.31, 1.06), [b]*p*=0.076				
**Pneumonia aetiology by culture of blood or lung/pleural aspirate or PCR on lung/pleural aspirate**				
Age 2-11 months	N=540	N=184		
PCR or culture pneumococcal	8	8	16	
Not PCR or culture pneumococcal	532	176	684	0.33 (0.11, 1.03)
Proportion PCR or culture pneumococcal	0.015	0.043	708	
Age 12-23 months	N=515	N=81		
PCR or culture pneumococcal	22	4	26	
Not PCR or culture pneumococcal	493	77	570	0.86 (0.28, 3.52)
Proportion PCR or culture pneumococcal	0.043	0.049	596	
Age 2-4 years	N=230	N=218		
PCR or culture pneumococcal	13	21	34	
Not PCR or culture pneumococcal	217	197	414	0.56 (0.25, 1.21)
Proportion PCR or culture pneumococcal	0.057	0.096	448	
Combined age strata 2-59 months, [a]M-H age-stratified odds ratio = 0.54 (0.33, 0.90), [b]*p*=0.017				

[a]Mantel-Haenzel age-stratified odds ratio. [b]Fisher's exact *p*-value.
