# Supplementary material for: Aetiology of lobar pneumonia determined by multiplex molecular analyses of lung and pleural aspirate specimens in the Gambia: findings from population-based pneumonia surveillance
Source: BMJ Open. 2022 Mar 9;12(3):e056706. doi: 10.1136/bmjopen-2021-056706 (PMC8915295; doi:10.1136/bmjopen-2021-056706)
Supplement: Author's manuscript [file bmjopen-2021-056706.draft_revisions.pdf]

BMJ Open is committed to open peer review. As part of this commitment we make the peer review history of every article we publish publicly available.

When an article is published we post the peer reviewers' comments and the authors' responses online. We also post the versions of the paper that were used during peer review. These are the versions that the peer review comments apply to.

The versions of the paper that follow are the versions that were submitted during the peer review process. They are not the versions of record or the final published versions. They should not be cited or distributed as the published version of this manuscript.

BMJ Open is an open access journal and the full, final, typeset and author-corrected version of record of the manuscript is available on our site with no access controls, subscription charges or pay-per-view fees (http://bmjopen.bmj.com).

If you have any questions on BMJ Open's open peer review process please email info.bmjopen@bmj.com

BMJ Open

# Aetiology of Lobar Pneumonia Determined by Multiplex Molecular Analyses of Lung and Pleural Aspirate Specimens in The Gambia



| | |
|---|---|
| Journal: | *BMJ Open* |
| Manuscript ID | bmjopen-2021-056706 |
| Article Type: | Original research |
| Date Submitted by the Author: | 23-Aug-2021 |





| | |
|---|---|
| Complete List of Authors: | Mackenzie, Grant; Medical Research Council Unit The Gambia at the London School of Hygiene and Tropical Medicine, Disease Control and Elimination; Murdoch Childrens Research Institute, Infection and Immunity<br>McLellan, Jessica; Medical Research Council Unit The Gambia at London School of Hygiene & Tropical Medicine, Disease Control and Elimination; University of Calgary, Cumming School of Medicine<br>Machuka, Eunice; Medical Research Council Unit The Gambia at London School of Hygiene & Tropical Medicine, Disease Control and Elimination<br>Pathirana, Jayani; Medical Research Council Unit The Gambia at London School of Hygiene & Tropical Medicine, Disease Control and Elimination<br>Fombah, Augustin; Medical Research Council Unit The Gambia at London School of Hygiene & Tropical Medicine, Disease Control and Elimination<br>Abatan, Baderinwa; Medical Research Council Unit The Gambia at London School of Hygiene & Tropical Medicine, Disease Control and Elimination<br>Manjang, Ahmed; Medical Research Council Unit The Gambia at London School of Hygiene & Tropical Medicine, Disease Control and Elimination<br>Ndiaye, Malick; Medical Research Council Unit The Gambia at the London School of Hygiene and Tropical Medicine, Disease Control and Elimination<br>Hossain, Ilias; Medical Research Council Unit The Gambia at the London School of Hygiene and Tropical Medicine, Disease Control and Elimination<br>Greenwood, B.; London School of Hygiene and Tropical Medicine, Faculty of Infectious and Tropical Disease<br>Hill, Philip; University of Otago, Centre for International Health |





| | |
|---|---|
| Keywords: | Epidemiology < TROPICAL MEDICINE, Molecular diagnostics < INFECTIOUS DISEASES, Respiratory infections < THORACIC MEDICINE |



SCHOLARONE™
Manuscripts

*I, the Submitting Author has the right to grant and does grant on behalf of all authors of the Work (as defined in the below author licence), an exclusive licence and/or a non-exclusive licence for contributions from authors who are: i) UK Crown employees; ii) where BMJ has agreed a CC-BY licence shall apply, and/or iii) in accordance with the terms applicable for US Federal Government officers or employees acting as part of their official duties; on a worldwide, perpetual, irrevocable, royalty-free basis to BMJ Publishing Group Ltd ("BMJ") its licensees and where the relevant Journal is co-owned by BMJ to the co-owners of the Journal, to publish the Work in this journal and any other BMJ products and to exploit all rights, as set out in our licence.*

*The Submitting Author accepts and understands that any supply made under these terms is made by BMJ to the Submitting Author unless you are acting as an employee on behalf of your employer or a postgraduate student of an affiliated institution which is paying any applicable article publishing charge ("APC") for Open Access articles. Where the Submitting Author wishes to make the Work available on an Open Access basis (and intends to pay the relevant APC), the terms of reuse of such Open Access shall be governed by a Creative Commons licence – details of these licences and which Creative Commons licence will apply to this Work are set out in our licence referred to above.*

*Other than as permitted in any relevant BMJ Author's Self Archiving Policies, I confirm this Work has not been accepted for publication elsewhere, is not being considered for publication elsewhere and does not duplicate material already published. I confirm all authors consent to publication of this Work and authorise the granting of this licence.*

For peer review only

# Aetiology of Lobar Pneumonia Determined by Multiplex Molecular Analyses of Lung and Pleural Aspirate Specimens in The Gambia

Grant A Mackenzie[1,2,3,4] PhD, Jessica McLellan[1,5] PhD, Eunice Machuka[1] MSc, Malick Ndiaye[1] DP, Jayani Pathirana[1] PhD, Augustin Fombah[1] MD, Baderinwa Abatan[1] FWACP, Ilias Hossain[1] MPH, Ahmed Manjang[1] MSc, Brian Greenwood[3] MD, Philip C Hill[4] MD

1. Medical Research Council Unit The Gambia at London School of Hygiene & Tropical Medicine, Fajara, The Gambia, West Africa
2. Infection and Immunity Theme, Murdoch Children's Research Institute, Melbourne, Australia
3. Faculty of Infectious and Tropical Diseases, London School of Hygiene & Tropical Medicine, London, UK
4. Department of Paediatrics, University of Melbourne, Melbourne, Australia
5. Cumming School of Medicine, University of Calgary, Calgary, Canada
6. Centre for International Health, University of Otago, Dunedin, New Zealand

Keywords: Pneumonia; Aetiology; Lung Aspirate; Co-infection; Polymerase-chain-reaction; Gambia

Corresponding author: Dr Grant A Mackenzie, MRC Unit The Gambia at London School of Hygiene & Tropical Medicine, PO Box 273 Banjul, The Gambia, West Africa; ph. +220 7207826, email. gmackenzie@mrc.gm

Word count abstract – 250

Word count text – 2934



For peer review only - http://bmjopen.bmj.com/site/about/guidelines.xhtml

## Abstract

### Background

Pneumonia aetiology generally relies on insensitive blood cultures or an assumption that organisms in the pharynx are causal. We determined the causes of lobar pneumonia in rural Gambia using lung aspiration.

### Methods

Pneumonia surveillance was undertaken among all ages. Blood culture and chest radiographs were performed routinely while lung or pleural aspirates were collected from selected patients. 7-valent pneumococcal conjugate vaccine (PCV7) was introduced in August 2009 and replaced by PCV13 from May 2011. We used conventional microbiology, and from April 8, 2011 to July 17, 2012, utilized a multiplex PCR assay on lung aspirates. We calculated proportions with pathogens, associations between co-infecting pathogens, and PCV effectiveness.

### Results

2,550 patients were admitted with clinical pneumonia; 741 with lobar pneumonia or pleural effusion. We performed multiplex PCR on 156 lung and 4 pleural aspirates. Pathogens were detected in 116 specimens, *Streptococcus pneumoniae* (n=68), *Staphylococcus aureus* (n=26), and *Haemophilus influenzae* type b (n=11). Bacteria (n=97) were more common than viruses (n=49). Common viruses were bocavirus (n=11) and influenza (n=11). Co-infections were frequent (n=55). *M. catarrhalis* was detected in eight patients and in every case there was co-infection with *S. pneumoniae*. The odds ratio of vaccine-type pneumococcal pneumonia in patients with two or three compared to zero doses of PCV was 0.17 (95% CI 0.06, 0.51).

### Conclusions

Lobar pneumonia in rural Gambia was caused primarily by bacteria, particularly *S. pneumoniae* and *S. aureus*. Co-infection was common and *M. catarrhalis* always co-infected with *S. pneumoniae*. PCV was highly efficacious against vaccine-type pneumococcal pneumonia.




For peer review only - http://bmjopen.bmj.com/site/about/guidelines.xhtml

**Strengths and limitations of this study**

- We conducted population-based pneumonia surveillance and collected gold standard specimens directly from the infected lung to determine the aetiology of lobar pneumonia in 160 patients in rural Gambia.
- We used multiplex real-time quantitative PCR to detect up to 31 pathogens (excluding *Legionella*, *Klebsiella*, and *Mycobacterium tuberculosis*) in lung specimens.
- *Streptococcus pneumoniae* and *Staphylococcus aureus* were the predominant causes of lobar pneumonia. Bacterial-bacterial and bacterial-viral co-infections were common.
- Pneumococcal conjugate vaccine effectively prevented non-bacteraemic pneumococcal pneumonia.
- Our results are generalisable to patients with lobar pneumonia and not all patients with clinical pneumonia.

For peer review only



For peer review only - http://bmjopen.bmj.com/site/about/guidelines.xhtml

## INTRODUCTION

Most studies of the aetiology of pneumonia rely on either the insensitive culture of bacteria from blood or the non-specific detection of organisms in sputum or pharynx. Case-control studies have compared the prevalence of organisms in the pharynx of children with pneumonia and matched controls, relying on the assumption that organisms detected in the pharynx are also present and pathogenic in the lung.[1-4] The multi-site Pneumonia Etiology for Research in Child Health (PERCH) study extended these methods, combining conventional and molecular microbiology data from the pharynx, blood, and lung with an analytic approach to estimate the probability of specific aetiologies.[2]

Historic studies using lung aspirate specimens and conventional microbiology commonly found *S. pneumoniae* and *H. influenzae* to be the most frequent causes of lobar pneumonia.[5-8] More recent studies using lung aspirates have been uncommon. A Gambian study employing molecular methods in 47 lung and nine pleural aspirates, and the PERCH study with 37 lung and 15 pleural aspirates, identified a pneumococcal aetiology in 87% and 25% of patients respectively.[2;9] Co-infection was present in 51% [9] and 17% of patients respectively.[2;9] The PERCH study may have underestimated the prevalence of bacterial infection in pneumonia due to the inclusion of children with bronchiolitis, challenges enrolling very sick children, and an assumption that organisms in pharyngeal specimens correlate with the cause of pneumonia.[10]

The importance of determining the aetiology of pneumonia, particularly the role of co-infections and the impact of vaccination strategies, remains. We studied these questions in rural Gambia during the introduction of pneumococcal conjugate vaccination (PCV), applying conventional and molecular methods to lung specimens.

For peer review only

## METHODS

### Setting

This study was nested within a surveillance study for suspected pneumonia, septicaemia, or meningitis in the Basse and Fuladu West Health and Demographic Surveillance Systems (BHDSS and FWHDSS) in rural Gambia, which in January 2012, included approximately 170 043 and 89 389 residents respectively. Child mortality in the BHDSS in 2011 was 68 per 1000 live births. Surveillance commenced in the BHDSS on May 12, 2008 and in the FWHDSS on September 12, 2011. PCV7 was introduced on August 19, 2009 and replaced by PCV13 during May 2011.

### Patients and procedures

The surveillance has been described previously.[11] Standardized methods were used for detection of possible cases of pneumonia, septicaemia, meningitis, referral and clinical investigation.[12;13] Suspected pneumonia was defined as a history of cough or difficulty breathing with the presence of any one of the following: respiratory rate ≥40 or ≥50 per minute for children aged greater than or less than 12 months respectively, lower-chest-wall-indrawing, nasal flaring, grunting, oxygen saturation <92%, dullness to percussion, bronchial breathing or crackles on auscultation. Patients with suspected pneumonia had anthropometric measurements, peripheral oxygen saturation measured, blood cultured, and chest radiographs done. We did not test for HIV as this was not standard practice and prevalence in The Gambia is relatively low.[14] Chest radiographs were interpreted according to WHO recommendations[15] by two independent reviewers, with readings discordant for end-point consolidation (i.e. lobar pneumonia) resolved by a third reviewer. A percutaneous trans-thoracic lung or pleural fluid aspiration was considered if a pleural effusion or large, dense, peripheral pneumonic consolidation was present on radiograph, there were no contraindications, and the patient was clinically stable. Following written, informed consent, lung aspiration was performed by a clinician using aseptic technique with a 21 gauge needle, the sample diluted in 1 ml of sterile saline with an aliquot inoculated on culture media. Patients were observed for 4 hours post-procedure. Lung aspiration is established as a safe practice in The Gambia, with an excellent safety record and sensitivity as a diagnostic tool.[16] All patients admitted with clinical pneumonia from April 8,



For peer review only - http://bmjopen.bmj.com/site/about/guidelines.xhtml

2011 to July 17, 2012 were included in this study. We chose this period as it covered introduction period of PCV.

**Laboratory procedures**

Microbiological specimens were processed in Basse using conventional microbiological methods including staining of lung and pleural aspirates for *M. tuberculosis.*[17] Blood was cultured using an automated system (Bactec 9050, Beckton Dickinson, Belgium). We serotyped *S. pneumoniae* isolates by latex agglutination using factor and group-specific antisera (Statens Serum Institute, Copenhagen, Denmark).[18] *H. influenzae* isolates were serotyped by slide agglutination using polyvalent and monovalent antisera to types a, b, c, d, e and f (Beckton Dickinson, Erembodegem, Belgium). Isolates that did not agglutinate with polyvalent antisera were classified as non-typeable *H. influenzae*.

Total nucleic acid was extracted from a 200µl aliquot of lung and pleural aspirates (easyMAG, bioMériux, France) with an internal control. Extracts were subjected to quantitative multiplex PCR (Fast-track Diagnostics Resp-33 kit, Sliema, Malta) for a panel of 33 respiratory bacteria, fungi, and viruses (see Supplementary Material) with internal positive, and negative controls.[19] Standard PCR curves were derived from plasmid standards during the testing to calculate pathogen load from cycle threshold values. We did not use a density threshold to define a positive result based on the assumption that any putative pathogen detected in consolidated lung or pleural fluid is pathogenic and involved in the pneumonic process. Interpretation for some targets required combinations of results (see Supplementary Material). Assay specificity for the *Klebsiella pneumoniae* and *Legionella* sp. targets was poor and therefore results for these bacteria were omitted from analyses.

**Statistical analysis**

We summarised the characteristics of patients admitted with clinical pneumonia and classified them into three groups; no radiological lobar consolidation and lobar consolidation with or without lung or pleural aspirate. Categorical variables were assessed using chi-square tests and the Kruskal-Wallis test was used for



For peer review only - http://bmjopen.bmj.com/site/about/guidelines.xhtml

continuous variables. We calculated age-stratified proportions of patients with pathogens identified in lung or pleural aspirates using multiplex PCR. Values of pathogen quantity were transformed to $log_{10}$ copies per ml. We tabulated the frequency of co-infection by pairs of pathogens. We used test-negative analyses to estimate the effectiveness of PCV to prevent pneumococcal pneumonia and vaccine-type pneumococcal pneumonia; combining conventional culture and serotype results with PCR results as appropriate. We calculated the odds of a positive versus negative test for the outcome in patients who had received ≥2 doses of PCV compared to zero doses seven or more days before admission. We calculated odds ratios and 95% confidence intervals in crude and age-stratified analyses using the Mantel-Haenszel method. Fisher's exact *p*-values were used for hypothesis tests. Analyses were done using STATA version 16 (StataCorp, College Station, USA).

**Ethical considerations**

Ethical approval was granted for the study by the Gambia Government/Medical Research Council (UK) Joint Ethics Committee (numbers 1087 and 1247). Written informed consent was obtained from patients or guardian for all study procedures.

**Patient and public involvement**

Patients and public were not involved in the design and conduct of the primary surveillance study that generated the data analysed for this report. Reporting of the primary study results and dissemination of results included a joint press release by the Gambia Ministry of Health and MRCG at LSHTM, as well as local feedback to health authorities and local communities in the study area. Patients and public were not involved in the specific sub-analysis of pneumonia aetiology data presented in this manuscript.

## RESULTS

Over the 21 month study period, 2550 patients were hospitalized with clinical pneumonia; 2406 were aged 0-59 months and 141 were aged ≥5 years (figure 1). WHO-defined radiological pneumonia with consolidation (i.e. lobar pneumonia) was detected in 741 (29%) patients. Of those with lobar pneumonia,



For peer review only - http://bmjopen.bmj.com/site/about/guidelines.xhtml

lung or pleural aspirates were collected from 176 and five (24%, 181/741) patients respectively. There were no complications following the lung aspiration procedures. Patients with lobar pneumonia aged 0-60 days were less likely than older patients to have a lung aspirate (1/64 versus 180/681) while older children and adults were more likely to have a lung aspirate than children aged 2-59 months (44/89 versus 136/592) [table 1]. Bacteremia was more common in patients who had a lung aspirate (31/178, 17%) compared to those without a lung aspirate (113/2119, 5%).

**Table 1. Characteristics of 2,550 patients admitted to hospital with clinical pneumonia, radiological findings and investigation with lung or pleural aspiration**

| Characteristic | Sub-group | No lobar consolidation (N=1,646) | Lobar consolidation no lung/pleural aspirate (N=564) | Lobar consolidation & lung/pleural aspirate (N=181) |
|---|---|---|---|---|
| Age | 0-60 dy | 143 (8.7%) | 63 (11.2%) | 1 (0.5%) |
| | 2-59 mo | 1,452 (88.2%) | 456 (80.9%) | 136 (75.1%) |
| | 5-14 yr | 42 (2.6%) | 23 (4.1%) | 26 (14.4%) |
| | ≥15 yr | 9 (0.5%) | 22 (3.9%) | 18 (9.9%) |
| Male | | 933 (56.7%) | 324 (57.4%) | 104 (57.5%) |
| [a]Mean respiratory rate/min | | 57.3 | 61.6 | 60.8 |
| [a]Mean oxygen saturation % | | 95.8% | 93.6% | 95.1% |
| [a]Wheeze | | 319/1,641 (19.4%) | 76/562 (13.5%) | 11/181 (6.1%) |
| [b]Tachycardia | | 963 (58.5%) | 351 (62.2%) | 138 (76.2%) |
| [a]Temperature ≥38.0˚C | | 823 (50.0%) | 347 (61.5%) | 126 (69.6%) |
| [ac]Prostration | 0-59 mo | 116/1,572 (7.4%) | 34/513 (6.6%) | 5/136 (3.7%) |
| [ad]WfH z-score <-3 | 0-59 mo | 274/1,587 (17.3%) | 95/513 (18.5%) | 28/136 (20.6%) |
| [e]BMI grade 3 thinness | 5-17 yr | 9/44 (20%) | 8/28 (29%) | 8/28 (29%) |
| [e]BMI <18.5 | ≥18 yr | 1/7 (14%) | 4/16 (25%) | 3/15 (20%) |
| Blood culture taken | | 1,584 (96.2%) | 535 (94.9%) | 178 (98.3%) |
| Blood culture pathogen isolated | | 82/1,584 (5.2%) | 31/535 (5.8%) | 31/178 (17.4%) |
| [f]PCV immunisation doses | 0 | 357/1,452 (24.6%) | 109/456 (23.9%) | 43/136 (31.6%) |
| | 1 | 159/1,452 (11.0%) | 38/456 (8.3%) | 12/136 (8.8%) |
| | 2 | 152/1,452 (10.5%) | 50/456 (11.0%) | 10/136 (7.4%) |



For peer review only - http://bmjopen.bmj.com/site/about/guidelines.xhtml

| | | | | |
|---|---|---|---|---|
| | 3 | 784/1,452 (54.0%) | 259/456 (56.8%) | 71/136 (52.2%) |
| Died in hospital | | 65 (3.9%) | 25 (4.4%) | 6 (3.3%) |

Note: Column totals do not equal 2,550 as 159 patients did not have a chest radiograph.

[a]Missing values: respiratory rate (n=1), oxygen saturation (n=5), wheeze (n=7), temperature (n=1), weight (n=5), height (n=14), prostration (n=30).

[b]Tachycardia defined as heart rate at admission >160 bpm in infants 0-11 months, >150 bpm in children 12-23 months, >140 bpm in children 2-4 years, and >100 bpm in those aged ≥5 years.

[c]Prostration defined as inability to sit if usually able or inability to feed.

[d]WfH – weight for height.

[e]BMI – body mass index.

[f]PCV doses if age 2-59 months; PCV7 only (no consolidation [n=441], consolidation no LA/PA [n=156], consolidation LA/PA [n=58]), PCV13 only (no consolidation [n=300], consolidation no LA/PA [n=88], consolidation LA/PA [n=6]), PCV7 and PCV13 (no consolidation [n=195], consolidation no LA/PA [n=65], consolidation LA/PA [n=17]).

Multiplex PCR was performed on 160/181 lung and pleural aspirates. Twenty-one collected specimens were not stored or available for PCR analysis. Before the exclusion of *K. pneumoniae* and *Legionella* results due to poor specificity, at least one pathogen was detected in 132/160 patients, and after their exclusion, pathogens were detected in 116/160 (73%) lung specimens (lung and pleural aspirates combined), one pathogen in 61 (38%) and two or more in 55 (34%) [table 2]. Bacteria were detected in 97 (61%) specimens and viruses in 49 (31%). Bacteria only infections were detected in 67 (42%) and bacterial co-infections in 26 (16%) specimens. Viral only infections were detected in 18 (11%) specimens with bacterial-viral co-infections in 30 (19%).

**Table 2. Organisms identified by multiplex PCR assay in patients with lung (n=156) and pleural (n=4) aspirate specimens**

| Specific pathogens isolated | 0-23 mo (N=77) n (%) | 2-4 yr (N=43) n (%) | ≥5 yr (N=40) n (%) | All ages (N=160) n (%) |
|---|---|---|---|---|
| *Streptococcus pneumoniae* | 26 (34) | 22 (51) | 20 (50) | 68 (42.5) |



For peer review only - http://bmjopen.bmj.com/site/about/guidelines.xhtml

| | | | | |
|---|---|---|---|---|
| *Staphylococcus aureus* | 15 (19) | 7 (16) | 4 (10) | 26 (16.3) |
| *Haemophilus influenzae* type b | 6 (8) | 5 (12) | 0 (0) | 11 (6.9) |
| *Pneumocystis jirovecii* | 8 (10) | 1 (2) | 1 (3) | 10 (6.3) |
| *Moraxella catarrhalis* | 3 (4) | 4 (9) | 1 (3) | 8 (5.0) |
| *Salmonella* species | 5 (6) | 1 (2) | 2 (5) | 8 (5.0) |
| *Bordetella pertussis* | 3 (4) | 3 (7) | 1 (3) | 7 (4.4) |
| *Haemophilus influenzae* non-type b | 2 (3) | 3 (7) | 1 (3) | 6 (3.8) |
| *Chlamydia pneumoniae* | 0 (0) | 2 (5) | 1 (3) | 3 (1.9) |
| *Mycoplasma pneumoniae* | 1 (1) | 0 (0) | 1 (3) | 2 (1.3) |
| Bocavirus | 7 (9) | 1 (2) | 3 (8) | 11 (6.9) |
| Parainfluenza 1 | 3 (4) | 3 (7) | 2 (5) | 8 (5.0) |
| Influenza C | 2 (3) | 3 (7) | 2 (5) | 7 (4.4) |
| Cytomegalovirus | 4 (5) | 2 (5) | 0 (0) | 6 (3.8) |
| Coronavirus HKU1 | 2 (3) | 0 (0) | 2 (5) | 4 (2.5) |
| Coronavirus 43 | 0 (0) | 4 (9) | 0 (0) | 4 (2.5) |
| Respiratory syncytial virus | 2 (3) | 1 (2) | 0 (0) | 3 (1.9) |
| Influenza A | 2 (3) | 0 (0) | 0 (0) | 2 (1.3) |
| Influenza B | 1 (1) | 0 (0) | 1 (3) | 2 (1.3) |
| Rhinovirus | 1 (1) | 0 (0) | 1 (3) | 2 (1.3) |
| Adenovirus | 1 (1) | 1 (2) | 0 (0) | 2 (1.3) |
| Human metapneumovirus | 2 (3) | 0 (0) | 0 (0) | 2 (1.3) |
| | | | | |
| **Pathogen(s) isolated** | | | | |
| Any pathogen | 52 (68) | 35 (81) | 29 (73) | 116 (72.5) |
| No pathogen | 25 (32) | 8 (19) | 11 (27) | 44 (27.5) |
| 1 pathogen | 25 (32) | 16 (37) | 20 (50) | 61 (38.1) |
| 2 pathogens | 14 (18) | 14 (33) | 5 (10) | 33 (20.6) |
| 3 pathogens | 9 (12) | 1 (2) | 3 (8) | 13 (8.1) |
| 4 or more pathogens | 4 (5) | 4 (9) | 1 (3) | 9 (5.6) |
| Bacterial pathogen(s) | 43 (56) | 30 (70) | 24 (60) | 97 (60.6) |
| Bacterial pathogen(s) only | 30 (39) | 20 (47) | 17 (43) | 67 (41.9) |
| Viral pathogen(s) | 23 (30) | 15 (35) | 11 (28) | 49 (30.6) |
| Viral pathogen(s) only | 9 (12) | 5 (12) | 4 (10) | 18 (11.3) |
| | | | | |
| **Co-infections isolated** | | | | |



For peer review only - http://bmjopen.bmj.com/site/about/guidelines.xhtml

For peer review only

| | | | | |
|---|---|---|---|---|
| Bacterial-bacterial co-detection | 11 (14) | 9 (21) | 6 (15) | 26 (16.3) |
| Bacterial-viral co-detection | 13 (17) | 10 (23) | 7 (18) | 30 (18.8) |
| Viral-viral co-detection | 6 (6) | 2 (5) | 0 (0) | 7 (4.4) |

Note: *H. influenzae* non-type b if *H. influenzae* target positive and Hib target negative; Hib if both targets positive.

The most frequent pathogens by multiplex PCR in lung specimens were *S. pneumoniae* (n=68, 43%), *S. aureus* (n=26, 16%), Hib (n=11, 7%), bocavirus (n=11, 7%), influenza viruses (n=11, 7%), *Pneumocystis jirovecii* (n=10, 6%), *Moraxella catarrhalis* (n=8, 5%), *Salmonella* sp. (n=8, 5%), and parainfluenza virus 1 (n=8, 5%) [table 2]. Respiratory syncytial virus (RSV) was detected in only three specimens. *S. pneumoniae* was more prevalent in patients aged ≥2 years (42/83, 51%) compared to children aged 0-23 months (26/77, 34%), odds ratio (OR) 2.01 (95% CI 1.01, 4.01). In contrast, *S. aureus* was more common in children aged <5 years (22/120, 18%) compared to older children and adults (4/40, 10%), OR 2.02 (95% CI 0.62, 8.58). Hib was restricted to children aged <5 years. *P. jirovecii* was more common in children aged 0-23 months (8/77, 10%) compared to patients aged ≥5 years (2/83, 2%), OR 4.75 (95% CI 0.90, 47.0).

Co-infection by pairs of pathogens is shown in table 3. *M. catarrhalis* was detected in eight patients and in every case there was co-infection with *S. pneumoniae* (8/68 with *S. pneumoniae* versus 0/92 without *S. pneumoniae*, *p*=0.0007). *B. pertussis* was detected in seven patients and in six there was co-infection with *S. pneumoniae* (6/68 with *S. pneumoniae* versus 1/92 without *S. pneumoniae*, *p*=0.018). These comparisons are subject to multiple testing of 54 pairs of pathogens.



For peer review only - http://bmjopen.bmj.com/site/about/guidelines.xhtml

**Table 3. Frequency of detection of pairs of pathogens identified by multiplex PCR assay in 156 lung and 4 pleural aspirate specimens**

| Pathogen | *S. pneumoniae* | *S. aureus* | [a]Hib | *P. jirovecii* | *M. catarrhalis* | *Salmonella* | *B. pertussis* | [b]Hi non-b | Bocavirus | [c]Influenza | Parainfluenza 1 |
|---|---|---|---|---|---|---|---|---|---|---|---|
| ***S. pneumoniae*** | 68 | | | | | | | | | | |
| ***S. aureus*** | 13 | 26 | | | | | | | | | |
| **Hib** | 3 | 2 | 11 | | | | | | | | |
| ***P. jirovecii*** | 5 | 1 | 0 | 10 | | | | | | | |
| ***M. catarrhalis*** | 8 | 4 | 2 | 0 | 8 | | | | | | |
| ***Salmonella*** | 5 | 3 | 1 | 2 | 1 | 8 | | | | | |
| ***B. pertussis*** | 6 | 3 | 0 | 1 | 2 | 2 | 7 | | | | |
| **Hi non-b** | 4 | 1 | ND | 1 | 2 | 0 | 0 | 6 | | | |
| **Bocavirus** | 6 | 2 | 0 | 1 | 1 | 2 | 0 | 1 | 11 | | |
| **Influenza** | 5 | 0 | 0 | 1 | 0 | 1 | 2 | 1 | 0 | 11 | |
| **Parainfluenza 1** | 5 | 1 | 0 | 0 | 0 | 0 | 0 | 0 | 1 | 0 | 8 |

[a]Hib – *H. influenzae* type b; [b]Hi non-b – non-type b *H. influenzae*; [c]Influenza – Influenza A, B, C.

For peer review only



For peer review only - http://bmjopen.bmj.com/site/about/guidelines.xhtml

Using lung aspirate PCR results, the proportion of children aged 2-59 months hospitalized with clinical pneumonia in whom *S. pneumoniae* was detected was lower among those who had received ≥2 doses of PCV compared to zero doses (table 4); age stratified OR 0.42 (95% CI 0.16, 1.05). Using a combination of culture and lung specimen PCR results, the proportion in whom *S. pneumoniae* was detected was less among those who had received ≥2 doses of PCV compared to zero doses (Supplementary Table 2); age-stratified OR 0.54 (95% CI 0.33, 0.90). Using culture and serotyping results, the proportion of children in whom vaccine-type pneumococci were isolated was significantly less among those who had received ≥2 doses of PCV compared to zero doses (table 4); age-stratified OR 0.17 (95% CI 0.06, 0.51).

**Table 4. Association of pneumococcal pneumonia with PCV vaccination status**

| **Pneumonia aetiology by PCR on lung/pleural aspirate** | **Number of PCV doses (PCV7 or PCV13)** | | **Total N** | **Odds ratio (95% CI)** |
|---|---|---|---|---|
| | ≥2 doses | 0 doses | | |
| Age 2-11 months | N=27 | N=11 | | |
| PCR pneumococcal | 4 | 4 | 8 | |
| PCR not pneumococcal | 23 | 7 | 30 | 0.30 (0.04, 2.16) |
| Proportion PCR pneumococcal | 0.15 | 0.36 | 38 | |
| Age 12-23 months | N=26 | N=3 | | |
| PCR pneumococcal | 12 | 2 | 14 | |
| PCR not pneumococcal | 14 | 1 | 15 | 0.43 (0.007, 9.5) |
| Proportion PCR pneumococcal | 0.46 | 0.66 | 29 | |
| Age 2-4 years | N=18 | N=23 | | |
| PCR pneumococcal | 7 | 13 | 20 | |
| PCR not pneumococcal | 11 | 10 | 21 | 0.49 (0.12, 2.03) |
| Proportion PCR pneumococcal | 0.39 | 0.57 | 41 | |
| Combined age strata 2-59 months, [a]M-H age-stratified odds ratio = 0.42 (0.16, 1.05), [b]*p*=0.062 | | | | |
| **Pneumonia aetiology by culture of blood or lung/pleural aspirate and pneumococcal serotyping** | | | | |
| Age 2-11 months | N=540 | N=184 | | |
| [c]Vaccine-type pneumococcal | 1 | 1 | 2 | |

For peer review only

| | | | | |
|---|---|---|---|---|
| Not vaccine-type pneumococcal | 539 | 183 | 722 | 0.34 (0.004, 26.8) |
| Proportion vaccine-type pneumococcal | 0.002 | 0.005 | 700 | |
| Age 12-23 months | N=515 | N=81 | | |
| [c]Vaccine-type pneumococcal | 3 | 2 | 5 | |
| Not vaccine-type pneumococcal | 512 | 79 | 591 | 0.23 (0.03, 2.82) |
| Proportion vaccine-type pneumococcal | 0.006 | 0.025 | 596 | |
| Age 2-4 years | N=230 | N=218 | | |
| [c]Vaccine-type pneumococcal | 2 | 13 | 15 | |
| Not vaccine-type pneumococcal | 228 | 205 | 427 | 0.14 (0.02, 0.62) |
| Proportion vaccine-type pneumococcal | 0.009 | 0.059 | 441 | |
| Combined age strata 2-59 months, [a]M-H age-stratified odds ratio = 0.17 (0.06, 0.51), [b]*p*=0.0005 | | | | |

[a]Mantel-Haenzel age-stratified odds ratio. [b]Fisher's exact *p*-value. [c]Vaccine-type defined as PCV7 serotypes for children who received PCV7, and PCV13 serotypes for children who received PCV13 or a combination of PCV7 and PCV13.

The greatest pathogen load in lung specimens was associated with *S. pneumoniae* (median 5.34 [IQR 3.73, 6.24] $log_{10}$ copies/ml), *H. influenzae* non-type b (median 6.07 [IQR 5.32, 6.86] $log_{10}$ copies/ml) and parainfluenza virus (PIV) 1 (median 6.46 [IQR 4.74, 10.93] $log_{10}$ copies/ml) positive specimens (Supplementary Table 1). Low pathogen load was associated with *S. aureus* (median 2.15 [IQR 1.68, 4.14] $log_{10}$ copies/ml), bocavirus (median 2.77 [IQR 2.19, 3.40] $log_{10}$ copies/ml]), and cytomegalovirus (2.57 [IQR 2.38, 3.71] $log_{10}$ copies/ml) positive specimens.



For peer review only - http://bmjopen.bmj.com/site/about/guidelines.xhtml

**DISCUSSION**

We have investigated the aetiology of lobar pneumonia in rural West Africa by applying multiplex molecular methods to a large number of lung specimens. Pathogens were detected in 73% of specimens with bacteria predominant. *S. pneumoniae* (43%) was the dominant pathogen followed by *S. aureus* (16%). Co-infection was common (34%) with bacterial-bacterial co-infection similar in prevalence to bacterial-viral co-infection. We observed correlated co-infection between *M. catarrhalis* and *S. pneumoniae*. The estimated effectiveness of ≥2 doses of PCV to prevent vaccine-type pneumococcal pneumonia was 83% (95% CI 49%, 94%). We have shown previously the association of the pneumococcus with severe lobar pneumonia in the study area.[13;20] Despite a well-established vaccination program, Hib was aetiologic in 9% of lobar pneumonia in young children. These cases may relate to disease before the age of immunization, delayed vaccine administration, waning immunity or unvaccinated migrants, but also continued transmission despite over 91% coverage of the three-dose schedule.[21] Although ongoing cases of culture-positive invasive Hib disease are documented in The Gambia [23], it is only our attention to non-bacteraemic pneumonia that revealed this type of residual Hib disease.

The finding of *S. aureus* aetiology in 18% of lobar pneumonia cases in young children is of concern given that empiric therapy for severe pneumonia in our setting is penicillin/ampicillin and gentamicin,[22] which has sub-optimal activity against staphylococcus. Ceftriaxone is recommended for severely ill children with hypoxia, heart failure, or who are unable to feed. Cloxacillin is recommended if no improvement in 48 hours or staphylococcal pneumonia is suspected.[22] Unfortunately, clinical features indicative of staphylococcal pneumonia are not reliable and radiology and microbiology are not generally available. The finding of *P. jirovecii* in 10% of lobar pneumonia in 0-23 month-olds was surprising as HIV prevalence is low in our setting. This relatively high prevalence may relate to undiagnosed HIV, HIV exposure, malnutrition, or be related to chance with small numbers of cases (n=10). Additional data are needed before a recommendation for HIV



For peer review only - http://bmjopen.bmj.com/site/about/guidelines.xhtml

testing in children with lobar pneumonia is considered in this setting. We found *M. catarrhalis*, *Salmonella* sp., *B. pertussis*, and non-type b *H. influenzae* aetiologic in 4-5% of cases of lobar pneumonia.

We did not expect to find bocavirus as the most prevalent virus associated with lobar pneumonia (11/160), although our data are consistent with parainfluenza and influenza viruses causing severe lower respiratory infections. The PERCH study found RSV to be the virus most associated with severe pneumonia, and bocavirus as the 7$^{th}$ most associated virus.[2] However, bocavirus is a documented cause of pneumonia in The Gambia[9] and South Africa.[4] The single-site nature of our study and multi-country PERCH data, or temporal transmission during the period of our study, may explain the differences in the prevalence of bocavirus and RSV. Alternatively, differing mechanisms of disease may explain our low prevalence of RSV, causing primarily upper respiratory and bronchiolar infection without alveolar consolidation, and bocavirus causing parenchymal disease.

Our finding that bacteria dominate the aetiology of lobar pneumonia aligns with both historical studies using lung aspirates[5;7;8;23] and recent studies using lung aspirates and molecular detection methods.[2;9] A Gambian study from 2007-2009 investigated 53 lung and pleural aspirates and found *S. pneumoniae* in 48, *H. influenzae* in 12, *S. aureus* and *Acinetobacter* sp. in three each and only one virus only infection. RSV, adenovirus, and bocavirus were detected in co-infection in two cases each.[9] PERCH data from 2012-2013, in which PCR detected pathogens in 43% of 37 lung and 15 pleural aspirates, detected pneumococcus in 13 specimens, *S. aureus* in seven, Hib in four, *M. catarrhalis* in four, viruses in three, and no RSV.[2]

Our observation of co-infection with two (21%), three (8%), and four or more pathogens (6%) underscores the polymicrobial nature of lobar pneumonia. Bacterial-bacterial and bacterial-viral co-infections were of similar prevalence. In the setting of co-infection, the estimation of aetiological



For peer review only - http://bmjopen.bmj.com/site/about/guidelines.xhtml

proportions due to individual pathogens remains a challenge with all aetiological pathogens necessarily contributing to more than 100% of cases. The importance of co-infections, temporal pathogenesis, and the interplay of viral upper and bacterial lower respiratory infections, raises the potential for vaccine interventions to impact pathogenesis involving non-target pathogens. The synergistic role of *S. pneumoniae* has already been demonstrated in a vaccine probe study showing the administration of PCV prevented hospitalization with viral-associated lower respiratory disease.[24]

The correlation we observed between *M. catarrhalis* and *S. pneumoniae* is intriguing. This may be explained by true synergism or by correlation alone given these organisms commonly co-colonize the upper respiratory tract. Aspiration of upper respiratory flora in the pathogenesis of lobar pneumonia would result in co-detection of such bacteria in lung tissue, if bacteria were able to avoid neutrophil killing and other clearance mechanisms.

We estimated the effectiveness of PCV against non-bacteraemic pneumococcal pneumonia, which has not been possible in most trials. Among adults in the Netherlands the efficacy of one dose of PCV13 was 45% to prevent non-invasive vaccine-type pneumococcal pneumonia and 75% to prevent vaccine-type invasive disease. Our estimates of PCV effectiveness against vaccine-type (OR 0.17; 95% CI 0.06, 0.51) and all pneumococcal pneumonia (OR 0.42; 95% CI 0.16, 1.05) are similar to the Gambian PCV9 trial estimates of efficacy against lung aspirate positive vaccine-type (73%) and all pneumococcal pneumonia (68%).[25]

The main strength of our study is the inclusion of a significant number of lung aspirate specimens combined with a sensitive and specific multiplex PCR assay. Our study was limited by the range of potential pathogens detected and sample size. The multiplex assay excluded measles and *M. tuberculosis*. The PERCH study found no cases of *M. tuberculosis* in lung or pleural aspirates but it

For peer review only

was isolated in The Gambia in 7/255 induced sputum specimens.[2] The already cited Gambian study of 53 lung specimens found no cases of *M. tuberculosis.*[9] Our analyses excluded *Legionella* and *Klebsiella* sp. due to poor assay specificity. Our cross-sectional design was not able to investigate the temporal aspects of pneumonia pathogenesis. The limited duration of our study may also introduce potential bias due to variation in the seasonal transmission of individual pathogens.

Understanding the contribution of less prevalent pathogens in lobar pneumonia, the age distribution of pathogen aetiology, and questions concerning co-infection and synergism will require larger sample sizes. More sensitive and specific multiplex assays may identify additional pathogens. Studies of pneumonia aetiology, and childhood pneumonia in general, should carefully consider the use of specific case definitions, for example separating bronchiolitis and lobar pneumonia phenotypes, to avoid heterogeneity in outcome measurements.[26] Longitudinal studies of pneumonia pathogenesis, or vaccine probe studies (such as with an RSV vaccine), may help determine the relationships between viruses and bacteria. Studies of pathogen gene expression in the lung[27] may reveal new therapeutic approaches.

Our study provides important information concerning the aetiology of lobar pneumonia in a setting with significant child mortality during the period of introduction of PCV. Our findings may not be generalizable to settings with different levels of vaccine coverage and nasopharyngeal bacterial carriage. Further studies using lung aspirates will address a number of remaining important questions.



For peer review only - http://bmjopen.bmj.com/site/about/guidelines.xhtml

**Author affiliations**

See title page.

**Acknowledgements**

We are grateful to the staff at Basse District Hospital and Bansang Hospital and staff of the Pneumococcal Surveillance Project who provided clinical evaluation and care for the patients. The Regional Health Teams in Upper and Central River Region provided logistic support.

**Contributors**

GM conceived and designed the study, conducted the analysis, and wrote the first draft of the manuscript. JM and EM conducted multiplex qPCR analyses and reviewed the manuscript. MN, JP, AF, BA, and IH enrolled the patients, collected the specimens and reviewed the manuscript. AM conducted conventional microbiological analyses and reviewed the manuscript. BG and PH advised on analysis and reviewed the manuscript. All authors approved the final version of the manuscript for submission.

**Funding**

This work was supported by the Bill & Melinda Gates Foundation [grant number OPP1020327] and the Medical Research Council Unit The Gambia at London School of Hygiene & Tropical Medicine.

**Competing interests**

The authors declare no competing interests.

**Patient consent for publication**

Not required.

**Ethics approval**

Ethical approval was granted for the study by the Gambia Government/Medical Research Council (UK) Joint Ethics Committee (numbers 1087 and 1247). Written informed consent was obtained from patients or guardian for all study procedures.

**Provenance and peer review**



For peer review only - http://bmjopen.bmj.com/site/about/guidelines.xhtml

No part of this work has been written by a medical writer. Some of the findings of this study were presented at the 66th annual meeting of the American Society of Tropical Medicine & Hygiene (abstract #: 17-A-1389). A copy of the originally submitted manuscript was uploaded to the medRxiv preprint website; https://medrxiv.org/cgi/content/short/2021.07.02.21259855v1.

**Data availability statement**

Data are available upon reasonable request to the MRCG Scientific Coordinating Committee and Gambia Government/MRCG Joint Ethics Committee. Deidentified patient data may be requested from the MRCG Data Management and Archives department.

**ORCID ID**

Grant Mackenzie 0000-0002-4994-2627

Jessica McLellan 0000-0002-5486-9620

Eunice Machuka 0000-0002-1142-3442

Malick Ndiaye not available

Jayani Pathirana 0000-0001-9678-4759

Augustin Fombah not available

Baderinwa Abatan not available

Ilias Hossain 0000-0002-0571-4702

Ahmed Manjang 0000-0003-4475-3090

Brian Greenwood 0000-0001-5725-9118

Philip C Hill 0000-0002-7006-0549

**Exclusive licence**

I, the Submitting Author have the right to grant and do grant on behalf of all authors of the Work (as defined in the below author licence), an exclusive licence and/or a non-exclusive licence for contributions from authors who are: i) UK Crown employees; ii) where BMJ has agreed a CC-BY licence shall apply, and/or iii) in accordance with the terms applicable for US Federal Government



For peer review only - http://bmjopen.bmj.com/site/about/guidelines.xhtml

officers or employees acting as part of their official duties; on a worldwide, perpetual, irrevocable, royalty-free basis to BMJ Publishing Group Ltd ("BMJ") its licensees and where the relevant Journal is co-owned by BMJ to the co-owners of the Journal, to publish the Work in any BMJ product and to exploit all rights, as set out in our licence.

**Figure legends**

**Figure 1. Study profile**

For peer review only

**References**

(1) Selwyn BJ. The epidemiology of acute respiratory tract infection in young children: comparison of findings from several developing countries. Coordinated Data Group of BOSTID Researchers. *Rev Infect Dis* 1990;12:S870-88.

(2) PERCH Study Group. Causes of severe pneumonia requiring hospital admission in children without HIV infection from Africa and Asia: the PERCH multi-country case-control study. *Lancet* 2019;394:757-79.

(3) Benet T, Sanchez P, V, Messaoudi M, *et al*. Microorganisms Associated With Pneumonia in Children <5 Years of Age in Developing and Emerging Countries: The GABRIEL Pneumonia Multicenter, Prospective, Case-Control Study. *Clin Infect Dis* 2017;65:604-12.

(4) Zar HJ, Barnett W, Stadler A, Gardner-Lubbe S, Myer L, Nicol MP. Aetiology of childhood pneumonia in a well vaccinated South African birth cohort: a nested case-control study of the Drakenstein Child Health Study. *Lancet Respir Med* 2016;4:463-72.

(5) Shann F, Gratten M, Germer S, Linnemann V, Hazlett D, Payne R. Aetiology of pneumonia in children in Goroka Hospital, Papua New Guinea. *Lancet* 1984;2:537-41.

(6) Kalra SK, Sasidharan T, Vatwani V, Sarkar P. Lung puncture: a diagnostic aid in childhood pneumonia. *Indian Pediatr* 1981;18:727-30.

(7) Wall RA, Corrah PT, Mabey DC, Greenwood BM. The etiology of lobar pneumonia in the Gambia. *Bull World Health Organ* 1986;64:553-8.

(8) Silverman M, Stratton D, Diallo A, Egler LJ. Diagnosis of acute bacterial pneumonia in Nigerian children. Value of needle aspiration of lung. *Arch Dis Child* 1977;52:925-31.

(9) Howie SR, Morris GA, Tokarz R, *et al*. Etiology of severe childhood pneumonia in the Gambia, West Africa, determined by conventional and molecular microbiological analyses of lung and pleural aspirate samples. *Clin Infect Dis* 2014;59:682-5.

(10) Duke T. What the PERCH study means for future pneumonia strategies. *Lancet* 2019;394:714-6.



For peer review only - http://bmjopen.bmj.com/site/about/guidelines.xhtml

(11) Mackenzie GA, Plumb ID, Sambou S, *et al*. Monitoring the introduction of pneumococcal conjugate vaccines into West Africa: design and implementation of a population-based surveillance system. *PLoS Med* 2012;9:e1001161.

(12) Mackenzie GA, Hill PC, Jeffries DJ, *et al*. Effect of the introduction of pneumococcal conjugate vaccination on invasive pneumococcal disease in The Gambia: a population-based surveillance study. *Lancet Infect Dis* 2016;16:703-11.

(13) Mackenzie GA, Hill PC, Sahito SM, *et al*. Impact of the introduction of pneumococcal conjugate vaccination on pneumonia in The Gambia: population-based surveillance and case-control studies. *Lancet Infect Dis* 2017;17:965-73.

(14) National AIDS Control Programme. Republic of The Gambia, HIV Sentinel Surveillance Report 2011. Banjul: Ministry of Health & Social Welfare; 2012.

(15) World Health Organization Pneumonia Vaccine Trial Investigators' Group. Standardization of interpretation of chest radiographs for the diagnosis of pneumonia in children. World Health Organization, Department of Vaccines and Biologicals. 2001. Available at: http://www.who.int/vaccines-documents. Accessed 5 October 2004.

(16) Ideh RC, Howie SR, Ebruke B, *et al*. Transthoracic lung aspiration for the aetiological diagnosis of pneumonia: 25 years of experience from The Gambia. *Int J Tuberc Lung Dis* 2011;15:729-35.

(17) Adegbola RA, Falade AG, Sam BE, *et al*. The etiology of pneumonia in malnourished and well-nourished Gambian children. *Pediatr Infect Dis J* 1994;13:975-82.

(18) O'Neill KP, Lloyd-Evans N, Campbell H, Forgie IM, Sabally S, Greenwood BM. Latex agglutination test for diagnosing pneumococcal pneumonia in children in developing countries. *BMJ* 1989;298:1061-4.

(19) Driscoll AJ, Karron RA, Morpeth SC, *et al*. Standardization of Laboratory Methods for the PERCH Study. *Clin Infect Dis* 2017;64:S245-S252.

(20) Mackenzie GA, Hill PC, Jeffries DJ, *et al*. Impact of the introduction of pneumococcal conjugate vaccination on invasive pneumococcal disease and pneumonia in The Gambia: 10 years of population-based surveillance. *Lancet Infect Dis* 2021 [in press].

(21) Zaman SM, Howie SR, Ochoge M, *et al*. Impact of routine vaccination against Haemophilus influenzae type b in The Gambia: 20 years after its introduction. *J Glob Health* 2020;10:010416.

(22) Standard Treatment Guidelines.The Gambia: Ministry of Health; 2017.

(23) Diakparomre MA, Obi JO. Aetiological diagnosis of pneumonia in childhood by lung puncture. *Nigerian J of Paediatrics* 1981;8:61-4.

(24) Madhi SA, Klugman KP. A role for Streptococcus pneumoniae in virus-associated pneumonia. *Nat Med* 2004;10:811-3.

(25) Cutts FT, Zaman SM, Enwere G, *et al*. Efficacy of nine-valent pneumococcal conjugate vaccine against pneumonia and invasive pneumococcal disease in The Gambia: randomised, double-blind, placebo-controlled trial. *Lancet* 2005;365:1139-46.



For peer review only - http://bmjopen.bmj.com/site/about/guidelines.xhtml

(26) Mackenzie G. The definition and classification of pneumonia. *Pneumonia* 2016;8:14.

(27) Dunne EM, Hua Y, Salaudeen R, *et al*. Insights into pneumococcal pneumonia using lung aspirates and nasopharyngeal swabs collected from pneumonia patients in The Gambia. *J Infect Dis* **2020**; doi 10.1093/infdis/jiaa186.

For peer review only



For peer review only - http://bmjopen.bmj.com/site/about/guidelines.xhtml

**Figure 1. Study profile**

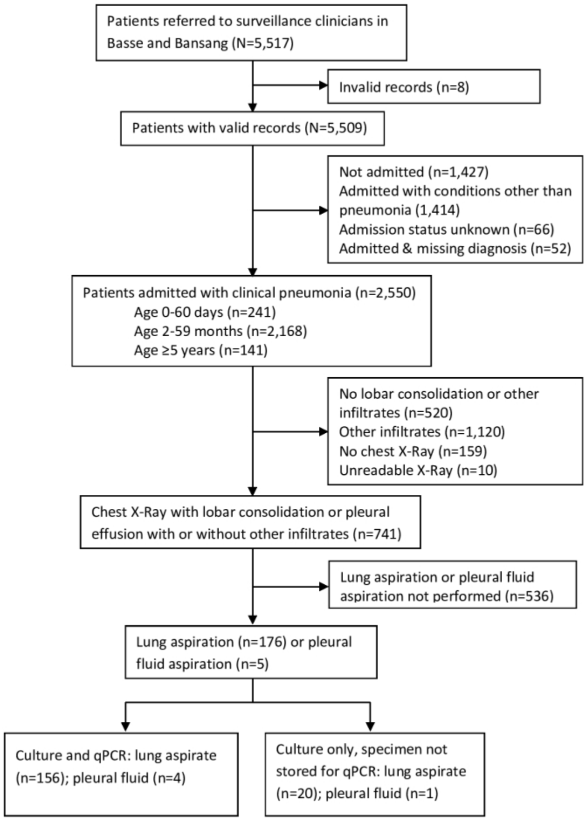


210x297mm (200 x 200 DPI)

# Aetiology of Lobar Pneumonia Determined by Multiplex Molecular Analyses of Lung and Pleural Aspirate Samples in The Gambia

***Methods for multiplex PCR assay***

Total nucleic acid was extracted from a 200µl aliquot of lung and pleural aspirates (easyMAG, bioMériux, France) with an internal control. Extracts were subjected to quantitative multiplex PCR (Fast-track Diagnostics Resp-33 kit, Sliema, Malta) for a panel of 33 respiratory bacteria, fungi, and viruses with internal positive and negative control. The assay was structured in eight component multiplex sub-assays with three or four targets run on one plate. We used a Bio-Rad CFX96 thermocycler with programming as recommended by the manufacturer. Standard PCR curves were derived from plasmid standards during the testing to calculate pathogen load from cycle threshold values. The multiplex PCR included the following targets:

- *S. pneumoniae* (lytA),
- *Haemophilus influenze* sp. (ompP6),
- *H. influenzae* type b (bexA),
- *S. aureus* (shkv),
- *Chlamydia pneumoniae* (RNApbc),
- *Moraxella catarrhalis* (copB),
- *Klebsiella pneumoniae* (khe),
- *Legionella* sp. (16SrRNA),
- *Pneumocystis jirovecii* (mtlsurRNA),
- *Bordetella pertussis* (is481),
- *Salmonella* sp. (ttrB),
- Influenza A (pos1), B (seg8ns1nep) and C (mtx),
- Cytomegalovirus (us7&8),
- Parainfluenza virus 1 (hnmRNA), 2 (hnmRNA), 3 (hnmRNA) and 4 (fus),
- Rhinovirus (utr),
- Coronaviruses NL63 (ncpn), 229E (ncpn), OC43 (ncpn) and HKU1 (ncpn),
- Respiratory syncytial virus A (nucap) and B (numRNA),
- Metapneumovirus A (fuglF) and B (fuglF),
- Adenovirus (hex),
- Bocavirus (np1),
- Enterovirus (dom4&5),
- Parechovirus (utr),
- *Mycoplasma pneumoniae* (adP1),

Data were not used for *K. pneumoniae* and Legionella spp. Interpretation for some targets required combinations of results: if rhinovirus only was detected then the specimen was deemed rhinovirus positive, whereas if rhinovirus and enterovirus were detected then the specimen was deemed enterovirus positive; if *H. influenzae* type b and *H. influenzae* were detected the specimen was deemed positive for *H. influenzae* type b, whereas if *H. influenzae* only was detected the specimen was deemed positive for *H. influenzae* non-type b.



For peer review only - http://bmjopen.bmj.com/site/about/guidelines.xhtml

***Clinical characteristics of patients***

Table 1 in the manuscript describes the characteristics of the patients in three categories: no lobar consolidation, lobar consolidation and no lung/pleural aspirate, and lobar consolidation and lung/pleural aspirate. Compared to patients without lobar pneumonia, those with lobar pneumonia had greater respiratory rate ($p$<0.0001), lower oxygen saturation ($p$=0.034), and less wheeze ($p$<0.0001), whereas heart rate ($p$=0.59), temperature ($p$=0.73), prostration ($p$=0.25), weight-for-height z-score <-3 in young children ($p$=0.28) and severe underweight in older children and adults ($p$=0.36) were not significantly different. Respiratory rate ($p$=0.50), heart rate ($p$=0.20), temperature ($p$=0.12), prostration ($p$=0.20), weight-for-height z-score <-3 in young children ($p$=0.62), and severe underweight in older children and adults ($p$=0.86) were not significantly different in patients with lobar pneumonia who did or did not have a lung aspirate, although wheeze was more frequent in patients without lung aspirate (76/562 versus 11/181, $p$=0.007) and oxygen saturation was greater ($p$=0.017). Bacteremia was more likely in patients who had a lung aspirate (31/178, 17%) compared to those without a lung aspirate, irrespective of whether lobar pneumonia was present on chest radiograph (113/2119, 5%). Ninety-six patients died (3.8%) with similar proportions in the three clinical categories.



For peer review only - http://bmjopen.bmj.com/site/about/guidelines.xhtml

***Quantification of pathogen load***

The greatest pathogen load in lung specimens was associated with *S. pneumoniae* (median 5.34 [IQR 3.73, 6.24] $log_{10}$ copies/ml), *H. influenzae* non-type b (median 6.07 [IQR 5.32, 6.86] $log_{10}$ copies/ml) and parainfluenza virus (PIV) 1 (median 6.46 [IQR 4.74, 10.93] $log_{10}$ copies/ml) positive specimens (Supplementary Table 1). Low pathogen load was associated with *S. aureus* (median 2.15 [IQR 1.68, 4.14] $log_{10}$ copies/ml), bocavirus (median 2.77 [IQR 2.19, 3.40] $log_{10}$ copies/ml]), and cytomegalovirus (2.57 [IQR 2.38, 3.71] $log_{10}$ copies/ml) positive specimens.

**Supplementary table 1. Organism-specific quantification of pathogen load in 156 lung and 4 pleural aspirate specimens**

| **Organism** | **Quantification of organism (median[IQR]; min, max); $log_{10}$ copies per ml** |
|---|---|
| **Bacteria** | |
| *Streptococcus pneumoniae* (n=68) | 5.34 (3.73 – 6.24); 1.44, 9.58 |
| *Staphylococcus aureus* (n=26) | 2.15 (1.68 – 4.14); 1.43, 8.49 |
| *Haemophilus influenzae* type b (n=11) | 4.18 (2.26 – 6.30); 1.56, 9.11 |
| *Moraxella catarrhalis* (n=8) | 4.40 (3.71 – 5.50); 2.63, 6.30 |
| *Salmonella* species (n=8) | 3.01 (1.74 – 5.29); 0.86, 9.07 |
| *Haemophilus influenzae* non-type b (n=6) | 6.07 (5.32 – 6.86); 4.88, 8.21 |
| *Bordetella pertussis* (n=4) | undef (undef); 0.30, 4.32 |
| *Chlamydia pneumonia* (n=3) | 3.60 (undef); 2.13, 4.73 |
| **Viruses** | |
| Bocavirus (n=11) | 2.77 (2.19 – 3.40); 1.53, 4.76 |
| Parainfluenza 1 (n=8) | 6.46 (4.74 – 10.93); 4.32, 12.50 |
| Influenza C (n=7) | 4.47 (4.21 – 5.64); 3.72, 6.85 |
| Cytomegalovirus (n=6) | 2.57 (2.38 – 3.71); 1.45, 5.89 |
| Coronavirus HKU1 (n=4) | 3.93 (undef); 3.77, 4.46 |
| Coronavirus 43 (n=4) | 4.77 (undef); 4.25, 5.37 |
| Respiratory syncytial virus (n=3) | 6.59 (undef); 5.07, 7.07 |
| **Fungi** | |
| *Pneumocystis jirovecii* (n=9) | 2.82 (2.52 – 3.37); 2.14, 7.42 |

Note: organisms listed were detected in three or more of 160 specimens. *B. pertussis* PCR Ct values were too great to allow quantification for three of seven specimens. The results of pathogen quantification in lobar pneumonia are subject to variation in the small volumes of specimen obtained and its dilution in 1ml of sterile saline.

For peer review only

***Effectiveness of PCV to prevent pneumococcal pneumonia***

**Supplementary table 2. Association of pneumococcal pneumonia with PCV vaccination status**

| **Pneumonia aetiology by culture of blood or lung/pleural aspirate** | **Number of PCV doses (PCV7 or PCV13)** | | **Total N** | **Odds ratio (95% CI)** |
|---|---|---|---|---|
| | ≥2 doses | 0 doses | | |
| Age 2-11 months | N=540 | N=184 | | |
| Culture pneumococcal | 5 | 5 | 10 | |
| Culture non-pneumococcal | 535 | 179 | 714 | 0.33 (0.08, 1.47) |
| Proportion culture pneumococcal | 0.009 | 0.027 | 724 | |
| Age 12-23 months | N=515 | N=81 | | |
| Culture pneumococcal | 15 | 2 | 17 | |
| Culture non-pneumococcal | 500 | 79 | 560 | 1.19 (0.27, 10.9) |
| Proportion culture pneumococcal | 0.029 | 0.025 | 577 | |
| Age 2-4 years | N=230 | N=218 | | |
| Culture pneumococcal | 9 | 15 | 24 | |
| Culture non-pneumococcal | 221 | 203 | 424 | 0.55 (0.21, 1.38) |
| Proportion culture pneumococcal | 0.039 | 0.069 | 448 | |
| Combined age strata 2-59 months, [a]M-H age-stratified odds ratio = 0.57 (0.31, 1.06), [b]*p*=0.076 | | | | |
| **Pneumonia aetiology by culture of blood or lung/pleural aspirate or PCR on lung/pleural aspirate** | | | | |
| Age 2-11 months | N=540 | N=184 | | |
| PCR or culture pneumococcal | 8 | 8 | 16 | |
| Not PCR or culture pneumococcal | 532 | 176 | 684 | 0.33 (0.11, 1.03) |
| Proportion PCR or culture pneumococcal | 0.015 | 0.043 | 708 | |
| Age 12-23 months | N=515 | N=81 | | |
| PCR or culture pneumococcal | 22 | 4 | 26 | |
| Not PCR or culture pneumococcal | 493 | 77 | 570 | 0.86 (0.28, 3.52) |
| Proportion PCR or culture pneumococcal | 0.043 | 0.049 | 596 | |
| Age 2-4 years | N=230 | N=218 | | |
| PCR or culture pneumococcal | 13 | 21 | 34 | |
| Not PCR or culture pneumococcal | 217 | 197 | 414 | 0.56 (0.25, 1.21) |
| Proportion PCR or culture pneumococcal | 0.057 | 0.096 | 448 | |
| Combined age strata 2-59 months, [a]M-H age-stratified odds ratio = 0.54 (0.33, 0.90), [b]*p*=0.017 | | | | |

[a]Mantel-Haenzel age-stratified odds ratio. [b]Fisher's exact *p*-value.



For peer review only - http://bmjopen.bmj.com/site/about/guidelines.xhtml

STROBE Statement—Checklist of items that should be included in reports of ***cross-sectional studies***

| | Item No | Recommendation | Page No |
|---|---|---|---|
| **Title and abstract** | 1 | (*a*) Indicate the study's design with a commonly used term in the title or the abstract | 1 |
| | | (*b*) Provide in the abstract an informative and balanced summary of what was done and what was found | 2 |
| **Introduction** | | | |
| Background/rationale | 2 | Explain the scientific background and rationale for the investigation being reported | 4 |
| Objectives | 3 | State specific objectives, including any prespecified hypotheses | 4 |
| **Methods** | | | |
| Study design | 4 | Present key elements of study design early in the paper | 5 |
| Setting | 5 | Describe the setting, locations, and relevant dates, including periods of recruitment, exposure, follow-up, and data collection | 5 |
| Participants | 6 | (*a*) Give the eligibility criteria, and the sources and methods of selection of participants | 5 |
| Variables | 7 | Clearly define all outcomes, exposures, predictors, potential confounders, and effect modifiers. Give diagnostic criteria, if applicable | 7 |
| Data sources/ measurement | 8* | For each variable of interest, give sources of data and details of methods of assessment (measurement). Describe comparability of assessment methods if there is more than one group | 6 |
| Bias | 9 | Describe any efforts to address potential sources of bias | 7 |
| Study size | 10 | Explain how the study size was arrived at | 8 |
| Quantitative variables | 11 | Explain how quantitative variables were handled in the analyses. If applicable, describe which groupings were chosen and why | 7 |
| Statistical methods | 12 | (*a*) Describe all statistical methods, including those used to control for confounding | 7 |
| | | (*b*) Describe any methods used to examine subgroups and interactions | 7 |
| | | (*c*) Explain how missing data were addressed | nd |
| | | (*d*) If applicable, describe analytical methods taking account of sampling strategy | 7 |
| | | (*e*) Describe any sensitivity analyses | nd |
| **Results** | | | |
| Participants | 13* | (a) Report numbers of individuals at each stage of study—eg numbers potentially eligible, examined for eligibility, confirmed eligible, included in the study, completing follow-up, and analysed | 8 |
| | | (b) Give reasons for non-participation at each stage | 8 |
| | | (c) Consider use of a flow diagram | 8 |
| Descriptive data | 14* | (a) Give characteristics of study participants (eg demographic, clinical, social) and information on exposures and potential confounders | 8 |
| | | (b) Indicate number of participants with missing data for each variable of interest | 9 |
| Outcome data | 15* | Report numbers of outcome events or summary measures | 9 |

For peer review only



For peer review only - http://bmjopen.bmj.com/site/about/guidelines.xhtml

| | | | |
|---|---|---|---|
| Main results | 16 | (*a*) Give unadjusted estimates and, if applicable, confounder-adjusted estimates and their precision (eg, 95% confidence interval). Make clear which confounders were adjusted for and why they were included | 11-13 |
| | | (*b*) Report category boundaries when continuous variables were categorized | na |
| | | (*c*) If relevant, consider translating estimates of relative risk into absolute risk for a meaningful time period | na |
| Other analyses | 17 | Report other analyses done—eg analyses of subgroups and interactions, and sensitivity analyses | 10 |
| **Discussion** | | | |
| Key results | 18 | Summarise key results with reference to study objectives | 15 |
| Limitations | 19 | Discuss limitations of the study, taking into account sources of potential bias or imprecision. Discuss both direction and magnitude of any potential bias | 17 |
| Interpretation | 20 | Give a cautious overall interpretation of results considering objectives, limitations, multiplicity of analyses, results from similar studies, and other relevant evidence | 18 |
| Generalisability | 21 | Discuss the generalisability (external validity) of the study results | 18 |
| **Other information** | | | |
| Funding | 22 | Give the source of funding and the role of the funders for the present study and, if applicable, for the original study on which the present article is based | 19 |

*Give information separately for exposed and unexposed groups.

**Note:** An Explanation and Elaboration article discusses each checklist item and gives methodological background and published examples of transparent reporting. The STROBE checklist is best used in conjunction with this article (freely available on the Web sites of PLoS Medicine at http://www.plosmedicine.org/, Annals of Internal Medicine at http://www.annals.org/, and Epidemiology at http://www.epidem.com/). Information on the STROBE Initiative is available at www.strobe-statement.org.

For peer review only

BMJ Open

# Aetiology of lobar pneumonia determined by multiplex molecular analyses of lung and pleural aspirate specimens in The Gambia: findings from population-based pneumonia surveillance

| | |
|---|---|
| Journal: | *BMJ Open* |
| Manuscript ID | bmjopen-2021-056706.R1 |
| Article Type: | Original research |
| Date Submitted by the Author: | 28-Jan-2022 |
| Complete List of Authors: | Mackenzie, Grant; Medical Research Council Unit The Gambia at the London School of Hygiene and Tropical Medicine, Disease Control and Elimination; Murdoch Childrens Research Institute, Infection and Immunity<br>McLellan, Jessica; Medical Research Council Unit The Gambia at London School of Hygiene & Tropical Medicine, Disease Control and Elimination; University of Calgary, Cumming School of Medicine<br>Machuka, Eunice; Medical Research Council Unit The Gambia at London School of Hygiene & Tropical Medicine, Disease Control and Elimination<br>Ndiaye, Malick; Medical Research Council Unit The Gambia at the London School of Hygiene and Tropical Medicine, Disease Control and Elimination<br>Pathirana, Jayani; Medical Research Council Unit The Gambia at London School of Hygiene & Tropical Medicine, Disease Control and Elimination<br>Fombah, Augustin; Medical Research Council Unit The Gambia at London School of Hygiene & Tropical Medicine, Disease Control and Elimination<br>Abatan, Baderinwa; Medical Research Council Unit The Gambia at London School of Hygiene & Tropical Medicine, Disease Control and Elimination<br>Hossain, Ilias; Medical Research Council Unit The Gambia at the London School of Hygiene and Tropical Medicine, Disease Control and Elimination<br>Manjang, Ahmed; Medical Research Council Unit The Gambia at London School of Hygiene & Tropical Medicine, Disease Control and Elimination<br>Greenwood, B.; London School of Hygiene and Tropical Medicine, Faculty of Infectious and Tropical Disease<br>Hill, Philip; University of Otago, Centre for International Health |
| <b>Primary Subject Heading</b>: | Infectious diseases |
| Secondary Subject Heading: | Epidemiology |
| Keywords: | Epidemiology < TROPICAL MEDICINE, Molecular diagnostics < INFECTIOUS DISEASES, Respiratory infections < THORACIC MEDICINE |
| | |

SCHOLARONE™
Manuscripts

*I, the Submitting Author has the right to grant and does grant on behalf of all authors of the Work (as defined in the below author licence), an exclusive licence and/or a non-exclusive licence for contributions from authors who are: i) UK Crown employees; ii) where BMJ has agreed a CC-BY licence shall apply, and/or iii) in accordance with the terms applicable for US Federal Government officers or employees acting as part of their official duties; on a worldwide, perpetual, irrevocable, royalty-free basis to BMJ Publishing Group Ltd ("BMJ") its licensees and where the relevant Journal is co-owned by BMJ to the co-owners of the Journal, to publish the Work in this journal and any other BMJ products and to exploit all rights, as set out in our licence.*

*The Submitting Author accepts and understands that any supply made under these terms is made by BMJ to the Submitting Author unless you are acting as an employee on behalf of your employer or a postgraduate student of an affiliated institution which is paying any applicable article publishing charge ("APC") for Open Access articles. Where the Submitting Author wishes to make the Work available on an Open Access basis (and intends to pay the relevant APC), the terms of reuse of such Open Access shall be governed by a Creative Commons licence – details of these licences and which Creative Commons licence will apply to this Work are set out in our licence referred to above.*

*Other than as permitted in any relevant BMJ Author's Self Archiving Policies, I confirm this Work has not been accepted for publication elsewhere, is not being considered for publication elsewhere and does not duplicate material already published. I confirm all authors consent to publication of this Work and authorise the granting of this licence.*

For peer review only

# Aetiology of lobar pneumonia determined by multiplex molecular analyses of lung and pleural aspirate specimens in The Gambia: findings from population-based pneumonia surveillance

Grant A Mackenzie[1,2,3,4] PhD, Jessica McLellan[1,5] PhD, Eunice Machuka[1] MSc, Malick Ndiaye[1] DP, Jayani Pathirana[1] PhD, Augustin Fombah[1] MD, Baderinwa Abatan[1] FWACP, Ilias Hossain[1] MPH, Ahmed Manjang[1] MSc, Brian Greenwood[3] MD, Philip C Hill[4] MD

1. Medical Research Council Unit The Gambia at London School of Hygiene & Tropical Medicine, Fajara, The Gambia, West Africa
2. Infection and Immunity Theme, Murdoch Children's Research Institute, Melbourne, Australia
3. Faculty of Infectious and Tropical Diseases, London School of Hygiene & Tropical Medicine, London, UK
4. Department of Paediatrics, University of Melbourne, Melbourne, Australia
5. Cumming School of Medicine, University of Calgary, Calgary, Canada
6. Centre for International Health, University of Otago, Dunedin, New Zealand

Keywords: Pneumonia; Aetiology; Lung Aspirate; Co-infection; Polymerase-chain-reaction; Gambia

Corresponding author: Dr Grant A Mackenzie, MRC Unit The Gambia at London School of Hygiene & Tropical Medicine, PO Box 273 Banjul, The Gambia, West Africa; ph. +220 7207826, email. gmackenzie@mrc.gm

Word count abstract – 273

Word count text – 3215

For peer review only

## Abstract

### Objectives

To determine the causes of lobar pneumonia in rural Gambia.

### Design and setting

Population-based pneumonia surveillance at seven peripheral health facilities and two regional hospitals in rural Gambia. 7-valent pneumococcal conjugate vaccine (PCV7) was introduced routinely in August 2009 and replaced by PCV13 from May 2011.

### Methods

Prospective pneumonia surveillance was undertaken among all ages with referral of suspected pneumonia cases to the regional hospitals. Blood culture and chest radiographs were performed routinely while lung or pleural aspirates were collected from selected, clinically stable patients with pleural effusion on radiograph and/or large, dense, peripheral consolidation. We used conventional microbiology, and from April 8, 2011 to July 17, 2012, utilized a multiplex PCR assay on lung and pleural aspirates. We calculated proportions with pathogens, associations between co-infecting pathogens, and PCV effectiveness.

### Participants

2,550 patients were admitted with clinical pneumonia; 741 with lobar pneumonia or pleural effusion. We performed 181 lung or pleural aspirates and multiplex PCR on 156 lung and 4 pleural aspirates.

### Results

Pathogens were detected in 116/160 specimens, *Streptococcus pneumoniae* (n=68), *Staphylococcus aureus* (n=26), and *Haemophilus influenzae* type b (n=11). Bacteria (n=97) were more common than viruses (n=49). Common viruses were bocavirus (n=11) and influenza (n=11). Co-infections were frequent (n=55). *M. catarrhalis* was detected in eight patients and in every case there was co-infection with *S. pneumoniae*. The odds ratio of vaccine-type pneumococcal pneumonia in patients with two or three compared to zero doses of PCV was 0.17 (95% CI 0.06, 0.51).

### Conclusions

For peer review only

Lobar pneumonia in rural Gambia was caused primarily by bacteria, particularly *S. pneumoniae* and *S. aureus*. Co-infection was common and *M. catarrhalis* always co-infected with *S. pneumoniae*. PCV was highly efficacious against vaccine-type pneumococcal pneumonia.

## Strengths and limitations of this study

- Population-based pneumonia surveillance collecting gold standard specimens directly from the infected lung to determine the aetiology of lobar pneumonia.
- Multiplex real-time quantitative PCR was used to detect up to 31 pathogens in lung specimens.
- However, multiplex PCR excluded *Legionella*, *Klebsiella*, and *Mycobacterium tuberculosis* and there was failure to detect a pathogen in 28% of patients with a lung specimen.
- Specific aetiology results and accurate vaccination records allowed calculation of the effectiveness of pneumococcal conjugate vaccine to prevent non-bacteraemic pneumococcal pneumonia.
- Results are generalisable to patients with lobar pneumonia, but not to all patients with clinical pneumonia.

## INTRODUCTION

The aetiology of childhood pneumonia is difficult to determine for a number of reasons: the upper respiratory tract is often colonised by pneumonia pathogens, a problem exacerbated with the use of overly sensitive molecular methods, the inability to produce sputum of good quality, and the difficulty obtaining a specimen from the alveolar space. Most studies of the aetiology of pneumonia rely on either the insensitive culture of bacteria from blood or the non-specific detection of organisms in sputum or pharynx. Case-control studies have compared the prevalence of organisms in the pharynx of children with pneumonia and matched controls, relying on the assumption that organisms detected in the pharynx are also present and pathogenic in the lung.[1-4] The multi-site Pneumonia Etiology for Research in Child Health (PERCH) study extended these methods, combining conventional and molecular microbiology data from the pharynx, blood, and lung with an analytic approach to estimate the probability of specific aetiologies.[2]



For peer review only - http://bmjopen.bmj.com/site/about/guidelines.xhtml

Historic studies using lung aspirate specimens and conventional microbiology commonly found *S. pneumoniae* and *H. influenzae* to be the most frequent causes of lobar pneumonia.[5-8] More recent studies using lung aspirates have been uncommon. A Gambian study employing molecular methods in 47 lung and nine pleural aspirates, and the PERCH study with 37 lung and 15 pleural aspirates, identified a pneumococcal aetiology in 87% and 25% of patients respectively.[2;9] Co-infection was present in 51% [9] and 17% of patients respectively.[2;9] The PERCH study may have underestimated the prevalence of bacterial infection in pneumonia due to the inclusion of children with bronchiolitis, challenges enrolling very sick children, and an assumption that organisms in pharyngeal specimens correlate with the cause of pneumonia.[10]

The importance of determining the aetiology of pneumonia, particularly the role of co-infections and the impact of vaccination strategies, remains. We studied these questions during the introduction of pneumococcal conjugate vaccination (PCV), applying conventional and molecular methods to lung specimens. We aimed to determine the aetiology of lobar pneumonia and the effectiveness of PCV to prevent pneumococcal pneumonia in rural Gambia.

## METHODS

### Setting

This study was nested within a population-based surveillance study for suspected pneumonia, septicaemia, or meningitis in the Basse and Fuladu West Health and Demographic Surveillance Systems (BHDSS and FWHDSS) in rural Gambia, which in January 2012, included approximately 170 043 and 89 389 residents respectively. Child mortality in the BHDSS in 2011 was 68 per 1000 live births. Surveillance commenced in the BHDSS on May 12, 2008 and in the FWHDSS on September 12, 2011. PCV7 was introduced on August 19, 2009 and replaced by PCV13 during May 2011.

### Patients and procedures



For peer review only - http://bmjopen.bmj.com/site/about/guidelines.xhtml

The surveillance has been described previously.[11] All patients presenting to the nine health facilities in the study area were screened 7 days per week, 24 hours per day, using standardized methods to detect possible cases of pneumonia, septicaemia, meningitis, referral and clinical investigation.[12;13] We used cross-sectional enrolment including all patients aged ≥2 months with suspected pneumonia. Suspected pneumonia was defined using modified WHO criteria, as a history of cough or difficulty breathing with the presence of any one of the following: respiratory rate ≥40 or ≥50 per minute for children aged greater than or less than 12 months respectively, lower-chest-wall-indrawing, nasal flaring, grunting, oxygen saturation <92%, dullness to percussion, bronchial breathing or crackles on auscultation. Patients with suspected pneumonia had anthropometric measurements, peripheral oxygen saturation measured, blood cultured, and chest radiographs done. We did not test for HIV as this was not standard practice and prevalence in The Gambia is relatively low.[14] Chest radiographs were interpreted according to WHO recommendations[15] by two independent reviewers, with readings discordant for end-point consolidation (i.e. lobar pneumonia) resolved by a third reviewer. A percutaneous trans-thoracic lung or pleural fluid aspiration was considered if a pleural effusion or large, dense, peripheral pneumonic consolidation was present on radiograph, there were no contraindications (post-measles pneumonia, pneumatocoeles on radiograph, skin sepsis, or no written informed consent), and the patient was clinically stable. Following written, informed consent, lung aspiration was performed by a clinician using aseptic technique with a 21 gauge needle and 5ml syringe with 1 ml of sterile saline with an aliquot inoculated on culture media. Specimens were immediately transported to the MRC Gambia, Basse laboratory, for preliminary analysis and stored at -80°C. Patients were observed for 3 hours post-procedure. Lung aspiration is established as a safe practice in The Gambia, with an excellent safety record and sensitivity as a diagnostic tool.[16] All patients admitted with clinical pneumonia from April 8, 2011 to July 17, 2012 were included in this study. We chose this period as it covered the introduction period of PCV.

**Laboratory procedures**

Microbiological specimens were processed in Basse using conventional microbiological methods including staining of lung and pleural aspirates for *M. tuberculosis.*[17] Blood was cultured using an automated system



For peer review only - http://bmjopen.bmj.com/site/about/guidelines.xhtml

(Bactec 9050, Beckton Dickinson, Belgium). Microbiological results were used to inform patient care. We serotyped *S. pneumoniae* isolates by latex agglutination using factor and group-specific antisera (Statens Serum Institute, Copenhagen, Denmark).[18] *H. influenzae* isolates were serotyped by slide agglutination using polyvalent and monovalent antisera to types a, b, c, d, e and f (Beckton Dickinson, Erembodegem, Belgium). Isolates that did not agglutinate with polyvalent antisera were classified as non-typeable *H. influenzae*.

Molecular analysis of lung specimens was conducted in two batches, in November/December 2011 and 2012, using the same methods, staff and laboratory as in the PERCH study in The Gambia. Total nucleic acid was extracted from a 200µl aliquot of lung and pleural aspirates (easyMAG, bioMériux, France) with an internal control. Extracts were subjected to quantitative multiplex PCR (Fast-track Diagnostics Resp-33 kit, Sliema, Malta) for a panel of 33 respiratory bacteria, fungi, and viruses (see Supplementary Material) with internal positive, and negative controls.[19] Standard PCR curves were derived from plasmid standards during the testing to calculate pathogen load from cycle threshold values. We did not use a density threshold to define a positive result based on the assumption that any putative pathogen detected in consolidated lung or pleural fluid is pathogenic and involved in the pneumonic process. Interpretation for some targets required combinations of results (see Supplementary Material). Assay specificity for the *Klebsiella pneumoniae* and *Legionella* sp. targets was poor and therefore results for these bacteria were omitted from analyses.

**Statistical analysis**

We summarised the characteristics of patients admitted with clinical pneumonia and classified them into three groups; no radiological lobar consolidation and lobar consolidation with or without lung or pleural aspirate. Categorical variables were assessed using chi-square tests and the Kruskal-Wallis test was used for continuous variables. We calculated age-stratified proportions of patients with pathogens identified in lung or pleural aspirates using multiplex PCR. Values of pathogen quantity were transformed to $\log_{10}$ copies per ml. We tabulated the frequency of co-infection by pairs of pathogens. We used test-negative analyses to



For peer review only - http://bmjopen.bmj.com/site/about/guidelines.xhtml

estimate the effectiveness of PCV to prevent pneumococcal pneumonia and vaccine-type pneumococcal pneumonia; combining conventional culture and serotype results with PCR results as appropriate. We calculated the odds of a positive versus negative test for the outcome in patients who had received ≥2 doses of PCV compared to zero doses seven or more days before admission. We calculated odds ratios and 95% confidence intervals in crude and age-stratified analyses using the Mantel-Haenszel method. Fisher's exact *p*-values were used for hypothesis tests. Analyses were done using STATA version 16 (StataCorp, College Station, USA).

For peer review only

**Ethical considerations**

Ethical approval was granted for the study by the Gambia Government/Medical Research Council (UK) Joint Ethics Committee (numbers 1087 and 1247). Written informed consent was obtained from patients or the parent/guardian for all study procedures. A separate written informed consent was obtained prior to each lung aspiration procedure.

**Patient and public involvement**

Patients and public were not involved in the design and conduct of the primary surveillance study that generated the data analysed for this report. Reporting of the primary study results and dissemination of results included a joint press release by the Gambia Ministry of Health and MRCG at LSHTM, as well as local feedback to health authorities and local communities in the study area. Patients and public were not involved in the specific sub-analysis of pneumonia aetiology data presented in this manuscript.

## RESULTS

Over the 21 month study period from April 8, 2011 to July 17, 2012, 2550 patients were hospitalized with clinical pneumonia; 2406 were aged 0-59 months and 141 were aged ≥5 years (figure 1). WHO-defined radiological pneumonia with consolidation (i.e. lobar pneumonia) was detected in 741 (29%) patients. Of those with lobar pneumonia, lung or pleural aspirates were collected from 176 and five (24%, 181/741) patients respectively. There were no complications following the lung aspiration procedures. Patients with



For peer review only - http://bmjopen.bmj.com/site/about/guidelines.xhtml

lobar pneumonia aged 0-60 days were less likely than older patients to have a lung aspirate (1/64 versus 180/681) while older children and adults were more likely to have a lung aspirate than children aged 2-59 months (44/89 versus 136/592) [table 1]. Bacteremia was more common in patients who had a lung aspirate (31/178, 17%) compared to those without a lung aspirate (113/2119, 5%).

**Table 1. Characteristics of 2,550 patients admitted to hospital with clinical pneumonia, radiological findings and investigation with lung or pleural aspiration**

| Characteristic | Sub-group | No lobar consolidation (N=1,646) | Lobar consolidation no lung/pleural aspirate (N=564) | Lobar consolidation & lung/pleural aspirate (N=181) |
|---|---|---|---|---|
| Age | 0-60 dy | 143 (8.7%) | 63 (11.2%) | 1 (0.5%) |
| | 2-59 mo | 1,452 (88.2%) | 456 (80.9%) | 136 (75.1%) |
| | 5-14 yr | 42 (2.6%) | 23 (4.1%) | 26 (14.4%) |
| | ≥15 yr | 9 (0.5%) | 22 (3.9%) | 18 (9.9%) |
| Male | | 933 (56.7%) | 324 (57.4%) | 104 (57.5%) |
| [a]Mean respiratory rate/min | | 57.3 | 61.6 | 60.8 |
| [a]Mean oxygen saturation % | | 95.8% | 93.6% | 95.1% |
| [a]Wheeze | | 319/1,641 (19.4%) | 76/562 (13.5%) | 11/181 (6.1%) |
| [b]Tachycardia | | 963 (58.5%) | 351 (62.2%) | 138 (76.2%) |
| [a]Temperature ≥38.0˚C | | 823 (50.0%) | 347 (61.5%) | 126 (69.6%) |
| [ac]Prostration | 0-59 mo | 116/1,572 (7.4%) | 34/513 (6.6%) | 5/136 (3.7%) |
| [ad]WfH z-score <-3 | 0-59 mo | 274/1,587 (17.3%) | 95/513 (18.5%) | 28/136 (20.6%) |
| [e]BMI grade 3 thinness | 5-17 yr | 9/44 (20%) | 8/28 (29%) | 8/28 (29%) |
| [e]BMI <18.5 | ≥18 yr | 1/7 (14%) | 4/16 (25%) | 3/15 (20%) |
| Blood culture taken | | 1,584 (96.2%) | 535 (94.9%) | 178 (98.3%) |
| Blood culture pathogen isolated | | 82/1,584 (5.2%) | 31/535 (5.8%) | 31/178 (17.4%) |
| [f]PCV immunisation doses | 0 | 357/1,452 (24.6%) | 109/456 (23.9%) | 43/136 (31.6%) |
| | 1 | 159/1,452 (11.0%) | 38/456 (8.3%) | 12/136 (8.8%) |
| | 2 | 152/1,452 (10.5%) | 50/456 (11.0%) | 10/136 (7.4%) |
| | 3 | 784/1,452 (54.0%) | 259/456 (56.8%) | 71/136 (52.2%) |
| Died in hospital | | 65 (3.9%) | 25 (4.4%) | 6 (3.3%) |

Note: Column totals do not equal 2,550 as 159 patients did not have a chest radiograph.



For peer review only - http://bmjopen.bmj.com/site/about/guidelines.xhtml

[a]Missing values: respiratory rate (n=1), oxygen saturation (n=5), wheeze (n=7), temperature (n=1), weight (n=5), height (n=14), prostration (n=30).

[b]Tachycardia defined as heart rate at admission >160 bpm in infants 0-11 months, >150 bpm in children 12-23 months, >140 bpm in children 2-4 years, and >100 bpm in those aged ≥5 years.

[c]Prostration defined as inability to sit if usually able or inability to feed.

[d]WfH – weight for height.

[e]BMI – body mass index.

[f]PCV doses if age 2-59 months; PCV7 only (no consolidation [n=441], consolidation no LA/PA [n=156], consolidation LA/PA [n=58]), PCV13 only (no consolidation [n=300], consolidation no LA/PA [n=88], consolidation LA/PA [n=6]), PCV7 and PCV13 (no consolidation [n=195], consolidation no LA/PA [n=65], consolidation LA/PA [n=17]).

Multiplex PCR was performed on 160/181 lung and pleural aspirates. Twenty-one collected specimens were not stored or available for PCR analysis. Before the exclusion of *K. pneumoniae* and *Legionella* results due to poor specificity, at least one pathogen was detected in 132/160 patients, and after their exclusion, pathogens were detected in 116/160 (73%) lung specimens (lung and pleural aspirates combined), one pathogen in 61 (38%) and two or more in 55 (34%) [table 2]. Bacteria were detected in 97 (61%) specimens and viruses in 49 (31%). Bacteria only infections were detected in 67 (42%) and bacterial co-infections in 26 (16%) specimens. Viral only infections were detected in 18 (11%) specimens with bacterial-viral co-infections in 30 (19%).

**Table 2. Organisms identified by multiplex PCR assay in patients with lung (n=156) and pleural (n=4) aspirate specimens**

| Specific pathogens isolated | 0-23 mo (N=77) n (%) | 2-4 yr (N=43) n (%) | ≥5 yr (N=40) n (%) | All ages (N=160) n (%) |
|---|---|---|---|---|
| *Streptococcus pneumoniae* | 26 (34) | 22 (51) | 20 (50) | 68 (42.5) |
| *Staphylococcus aureus* | 15 (19) | 7 (16) | 4 (10) | 26 (16.3) |
| *Haemophilus influenzae* type b | 6 (8) | 5 (12) | 0 (0) | 11 (6.9) |
| *Pneumocystis jirovecii* | 8 (10) | 1 (2) | 1 (3) | 10 (6.3) |
| *Moraxella catarrhalis* | 3 (4) | 4 (9) | 1 (3) | 8 (5.0) |



For peer review only - http://bmjopen.bmj.com/site/about/guidelines.xhtml

| | | | | |
|---|---|---|---|---|
| *Salmonella* species | 5 (6) | 1 (2) | 2 (5) | 8 (5.0) |
| *Bordetella pertussis* | 3 (4) | 3 (7) | 1 (3) | 7 (4.4) |
| *Haemophilus influenzae* non-type b | 2 (3) | 3 (7) | 1 (3) | 6 (3.8) |
| *Chlamydia pneumoniae* | 0 (0) | 2 (5) | 1 (3) | 3 (1.9) |
| *Mycoplasma pneumoniae* | 1 (1) | 0 (0) | 1 (3) | 2 (1.3) |
| Bocavirus | 7 (9) | 1 (2) | 3 (8) | 11 (6.9) |
| Parainfluenza 1 | 3 (4) | 3 (7) | 2 (5) | 8 (5.0) |
| Influenza C | 2 (3) | 3 (7) | 2 (5) | 7 (4.4) |
| Cytomegalovirus | 4 (5) | 2 (5) | 0 (0) | 6 (3.8) |
| Coronavirus HKU1 | 2 (3) | 0 (0) | 2 (5) | 4 (2.5) |
| Coronavirus 43 | 0 (0) | 4 (9) | 0 (0) | 4 (2.5) |
| Respiratory syncytial virus | 2 (3) | 1 (2) | 0 (0) | 3 (1.9) |
| Influenza A | 2 (3) | 0 (0) | 0 (0) | 2 (1.3) |
| Influenza B | 1 (1) | 0 (0) | 1 (3) | 2 (1.3) |
| Rhinovirus | 1 (1) | 0 (0) | 1 (3) | 2 (1.3) |
| Adenovirus | 1 (1) | 1 (2) | 0 (0) | 2 (1.3) |
| Human metapneumovirus | 2 (3) | 0 (0) | 0 (0) | 2 (1.3) |
| **Pathogen(s) isolated** | | | | |
| Any pathogen | 52 (68) | 35 (81) | 29 (73) | 116 (72.5) |
| No pathogen | 25 (32) | 8 (19) | 11 (27) | 44 (27.5) |
| 1 pathogen | 25 (32) | 16 (37) | 20 (50) | 61 (38.1) |
| 2 pathogens | 14 (18) | 14 (33) | 5 (10) | 33 (20.6) |
| 3 pathogens | 9 (12) | 1 (2) | 3 (8) | 13 (8.1) |
| 4 or more pathogens | 4 (5) | 4 (9) | 1 (3) | 9 (5.6) |
| Bacterial pathogen(s) | 43 (56) | 30 (70) | 24 (60) | 97 (60.6) |
| Bacterial pathogen(s) only | 30 (39) | 20 (47) | 17 (43) | 67 (41.9) |
| Viral pathogen(s) | 23 (30) | 15 (35) | 11 (28) | 49 (30.6) |
| Viral pathogen(s) only | 9 (12) | 5 (12) | 4 (10) | 18 (11.3) |
| **Co-infections isolated** | | | | |
| Bacterial-bacterial co-detection | 11 (14) | 9 (21) | 6 (15) | 26 (16.3) |
| Bacterial-viral co-detection | 13 (17) | 10 (23) | 7 (18) | 30 (18.8) |
| Viral-viral co-detection | 6 (6) | 2 (5) | 0 (0) | 7 (4.4) |

Note: *H. influenzae* non-type b if *H. influenzae* target positive and Hib target negative; Hib if both targets positive.



For peer review only - http://bmjopen.bmj.com/site/about/guidelines.xhtml

For peer review only

The most frequent pathogens by multiplex PCR in lung specimens were *S. pneumoniae* (n=68, 43%), *S. aureus* (n=26, 16%), Hib (n=11, 7%), bocavirus (n=11, 7%), influenza viruses (n=11, 7%), *Pneumocystis jirovecii* (n=10, 6%), *Moraxella catarrhalis* (n=8, 5%), *Salmonella* sp. (n=8, 5%), and parainfluenza virus 1 (n=8, 5%) [table 2]. Respiratory syncytial virus (RSV) was detected in only three specimens. *S. pneumoniae* was more prevalent in patients aged ≥2 years (42/83, 51%) compared to children aged 0-23 months (26/77, 34%), odds ratio (OR) 2.01 (95% CI 1.01, 4.01). In contrast, *S. aureus* was more common in children aged <5 years (22/120, 18%) compared to older children and adults (4/40, 10%), OR 2.02 (95% CI 0.62, 8.58). Hib was restricted to children aged <5 years. *P. jirovecii* was more common in children aged 0-23 months (8/77, 10%) compared to patients aged ≥5 years (2/83, 2%), OR 4.75 (95% CI 0.90, 47.0).

Co-infection by pairs of pathogens is shown in table 3. *M. catarrhalis* was detected in eight patients and in every case there was co-infection with *S. pneumoniae* (8/68 with *S. pneumoniae* versus 0/92 without *S. pneumoniae*, *p*=0.0007). *B. pertussis* was detected in seven patients and in six there was co-infection with *S. pneumoniae* (6/68 with *S. pneumoniae* versus 1/92 without *S. pneumoniae*, *p*=0.018). These comparisons are subject to multiple testing of 54 pairs of pathogens.

For peer review only

**Table 3. Frequency of detection of pairs of pathogens identified by multiplex PCR assay in 156 lung and 4 pleural aspirate specimens**

| Pathogen | *S. pneumoniae* | *S. aureus* | [a]Hib | *P. jirovecii* | *M. catarrhalis* | *Salmonella* | *B. pertussis* | [b]Hi non-b | Bocavirus | [c]Influenza | Parainfluenza 1 |
|---|---|---|---|---|---|---|---|---|---|---|---|
| ***S. pneumoniae*** | 68 | | | | | | | | | | |
| ***S. aureus*** | 13 | 26 | | | | | | | | | |
| **Hib** | 3 | 2 | 11 | | | | | | | | |
| ***P. jirovecii*** | 5 | 1 | 0 | 10 | | | | | | | |
| ***M. catarrhalis*** | 8 | 4 | 2 | 0 | 8 | | | | | | |
| ***Salmonella*** | 5 | 3 | 1 | 2 | 1 | 8 | | | | | |
| ***B. pertussis*** | 6 | 3 | 0 | 1 | 2 | 2 | 7 | | | | |
| **Hi non-b** | 4 | 1 | ND | 1 | 2 | 0 | 0 | 6 | | | |
| **Bocavirus** | 6 | 2 | 0 | 1 | 1 | 2 | 0 | 1 | 11 | | |
| **Influenza** | 5 | 0 | 0 | 1 | 0 | 1 | 2 | 1 | 0 | 11 | |
| **Parainfluenza 1** | 5 | 1 | 0 | 0 | 0 | 0 | 0 | 0 | 1 | 0 | 8 |

[a]Hib – *H. influenzae* type b; [b]Hi non-b – non-type b *H. influenzae*; [c]Influenza – Influenza A, B, C.

For peer review only

Using lung aspirate PCR results, the proportion of children aged 2-59 months hospitalized with clinical pneumonia in whom *S. pneumoniae* was detected was lower among those who had received ≥2 doses of PCV compared to zero doses (table 4); age stratified OR 0.42 (95% CI 0.16, 1.05). Using a combination of culture and lung specimen PCR results, the proportion in whom *S. pneumoniae* was detected was less among those who had received ≥2 doses of PCV compared to zero doses (Supplementary Table 2); age-stratified OR 0.54 (95% CI 0.33, 0.90). Using culture and serotyping results, the proportion of children in whom vaccine-type pneumococci were isolated was significantly less among those who had received ≥2 doses of PCV compared to zero doses (table 4); age-stratified OR 0.17 (95% CI 0.06, 0.51).

**Table 4. Association of pneumococcal pneumonia with PCV vaccination status**

| **Pneumonia aetiology by PCR on lung/pleural aspirate** | **Number of PCV doses (PCV7 or PCV13)** | | **Total N** | **Odds ratio (95% CI)** |
|---|---|---|---|---|
| | ≥2 doses | 0 doses | | |
| Age 2-11 months | N=27 | N=11 | | |
| Pneumococcal PCR +ve | 4 | 4 | 8 | |
| Pneumococcal PCR -ve | 23 | 7 | 30 | 0.30 (0.04, 2.16) |
| Proportion Pneumococcal PCR +ve | 0.15 | 0.36 | 38 | |
| Age 12-23 months | N=26 | N=3 | | |
| Pneumococcal PCR +ve | 12 | 2 | 14 | |
| Pneumococcal PCR -ve | 14 | 1 | 15 | 0.43 (0.007, 9.5) |
| Proportion Pneumococcal PCR +ve | 0.46 | 0.66 | 29 | |
| Age 2-4 years | N=18 | N=23 | | |
| Pneumococcal PCR +ve | 7 | 13 | 20 | |
| Pneumococcal PCR -ve | 11 | 10 | 21 | 0.49 (0.12, 2.03) |
| Proportion Pneumococcal PCR +ve | 0.39 | 0.57 | 41 | |
| Combined age strata 2-59 months, [a]M-H age-stratified odds ratio = 0.42 (0.16, 1.05), [b]$p$=0.062 | | | | |
| **Pneumonia aetiology by culture of blood or lung/pleural aspirate and pneumococcal serotyping** | | | | |
| Age 2-11 months | N=540 | N=184 | | |
| [c]Vaccine-type pneumococcal | 1 | 1 | 2 | |



For peer review only - http://bmjopen.bmj.com/site/about/guidelines.xhtml

| | | | | |
|---|---|---|---|---|
| Not vaccine-type pneumococcal | 539 | 183 | 722 | 0.34 (0.004, 26.8) |
| Proportion vaccine-type pneumococcal | 0.002 | 0.005 | 700 | |
| Age 12-23 months | N=515 | N=81 | | |
| [c]Vaccine-type pneumococcal | 3 | 2 | 5 | |
| Not vaccine-type pneumococcal | 512 | 79 | 591 | 0.23 (0.03, 2.82) |
| Proportion vaccine-type pneumococcal | 0.006 | 0.025 | 596 | |
| Age 2-4 years | N=230 | N=218 | | |
| [c]Vaccine-type pneumococcal | 2 | 13 | 15 | |
| Not vaccine-type pneumococcal | 228 | 205 | 427 | 0.14 (0.02, 0.62) |
| Proportion vaccine-type pneumococcal | 0.009 | 0.059 | 441 | |
| Combined age strata 2-59 months, [a]M-H age-stratified odds ratio = 0.17 (0.06, 0.51), [b]*p*=0.0005 | | | | |

[a]Mantel-Haenzel age-stratified odds ratio. [b]Fisher's exact *p*-value. [c]Vaccine-type defined as PCV7 serotypes for children who received PCV7, and PCV13 serotypes for children who received PCV13 or a combination of PCV7 and PCV13.

The greatest pathogen load in lung specimens was associated with *S. pneumoniae* (median 5.34 [IQR 3.73, 6.24] $log_{10}$ copies/ml), *H. influenzae* non-type b (median 6.07 [IQR 5.32, 6.86] $log_{10}$ copies/ml) and parainfluenza virus (PIV) 1 (median 6.46 [IQR 4.74, 10.93] $log_{10}$ copies/ml) positive specimens (Supplementary Table 1). Low pathogen load was associated with *S. aureus* (median 2.15 [IQR 1.68, 4.14] $log_{10}$ copies/ml), bocavirus (median 2.77 [IQR 2.19, 3.40] $log_{10}$ copies/ml]), and cytomegalovirus (2.57 [IQR 2.38, 3.71] $log_{10}$ copies/ml) positive specimens.


For peer review only - http://bmjopen.bmj.com/site/about/guidelines.xhtml

**DISCUSSION**

We have investigated the aetiology of lobar pneumonia in rural West Africa by applying multiplex molecular methods to a large number of lung specimens. Pathogens were detected in 73% of specimens with bacteria predominant. *S. pneumoniae* (43%) was the dominant pathogen followed by *S. aureus* (16%). Co-infection was common (34%) with bacterial-bacterial co-infection similar in prevalence to bacterial-viral co-infection. We observed correlated co-infection between *M. catarrhalis* and *S. pneumoniae*. The estimated effectiveness of ≥2 doses of PCV to prevent vaccine-type pneumococcal pneumonia was 83% (95% CI 49%, 94%). We have shown previously the association of the pneumococcus with severe lobar pneumonia in the study area.[13;20] Despite a well-established vaccination program, Hib was aetiologic in 9% of lobar pneumonia in young children. These cases may relate to disease before the age of immunization, delayed vaccine administration, waning immunity or unvaccinated migrants, but also continued transmission despite over 91% coverage of the three-dose schedule.[21] Although ongoing cases of culture-positive invasive Hib disease are documented in The Gambia,[21] it is only our attention to non-bacteraemic pneumonia that revealed this type of residual Hib disease.

The finding of *S. aureus* aetiology in 18% of lobar pneumonia cases in young children is of concern given that empiric therapy for severe pneumonia in our setting is penicillin/ampicillin and gentamicin,[22] which has sub-optimal activity against staphylococcus. Ceftriaxone is recommended for severely ill children with hypoxia, heart failure, or who are unable to feed. Cloxacillin is recommended if no improvement in 48 hours or staphylococcal pneumonia is suspected.[22] Unfortunately, clinical features indicative of staphylococcal pneumonia are not reliable and radiology and microbiology are not generally available. A review by Scott et al. of 33 studies from 1918-1997, based on LA culture, reported *S. aureus* in 15% of cases.[23] Studies analysing lung specimens using molecular methods report *S. aureus* in 3/53 specimens in The Gambia[9] and 7/37 specimens in PERCH[2]. The finding of *P. jirovecii* in 10% of lobar pneumonia in 0-23 month-olds was



For peer review only - http://bmjopen.bmj.com/site/about/guidelines.xhtml

surprising as HIV prevalence is low in our setting. This relatively high prevalence may relate to undiagnosed HIV, HIV exposure, malnutrition, or be related to chance with small numbers of cases (n=10). Additional data are needed before a recommendation for HIV testing in children with lobar pneumonia is considered in this setting. We found *M. catarrhalis*, *Salmonella* sp., *B. pertussis*, and non-type b *H. influenzae* aetiologic in 4-5% of cases of lobar pneumonia.

We did not expect to find bocavirus as the most prevalent virus associated with lobar pneumonia (11/160), although our data are consistent with parainfluenza and influenza viruses causing severe lower respiratory infections. The PERCH study found RSV to be the virus most associated with severe pneumonia, and bocavirus as the 7th most associated virus.[2] However, bocavirus is a documented cause of pneumonia in The Gambia[9] and South Africa.[4] The single-site nature of our study or variable seasonal transmission during the relatively short study period that included one wet season (typically the RSV season) and two dry seasons (typically low viral transmission) may explain the differences in the prevalence of bocavirus and RSV in our lung specimens. Delayed processing of specimens may relate to preferential detection of DNA (e.g. bocavirus) compared to RNA (e.g. RSV) viruses but this is unlikely given our close attention to specimen handling. Alternatively, the consistency of our data with the similar paucity of RSV detected in lung specimens in PERCH[2] and by Howie and colleagues,[9] suggest that differing mechanisms of disease may explain the low prevalence of RSV in lung specimens, with RSV causing primarily upper respiratory and bronchiolar infection without alveolar consolidation, and bocavirus causing parenchymal disease.

Our finding that bacteria dominate the aetiology of lobar pneumonia aligns with both historical studies using lung aspirates[5;7;8;24] and recent studies using lung aspirates and molecular detection methods.[2;9] A Gambian study from 2007-2009 investigated 53 lung and pleural aspirates and found *S. pneumoniae* in 48, *H. influenzae* in 12, *S. aureus* and *Acinetobacter* sp. in three each and only one virus only infection. RSV, adenovirus, and bocavirus were detected in co-infection in two cases each.[9]



For peer review only - http://bmjopen.bmj.com/site/about/guidelines.xhtml

PERCH data from 2012-2013, in which PCR detected pathogens in 43% of 37 lung and 15 pleural aspirates, detected pneumococcus in 13 specimens, *S. aureus* in seven, Hib in four, *M. catarrhalis* in four, viruses in three, and no RSV.[2] The predominance of bacteria in our data and the lung specimens of the PERCH study[2] and Howie and colleagues,[9] which differs from the viral preponderance in the hospitalised pneumonia cases in PERCH, appears to be related to a difference in clinical phenotype, with PERCH cases having WHO-defined endpoint consolidation and/or infiltrates on radiograph. Interestingly, the definition of cases in the GABRIEL study specified the presence of WHO-defined endpoint consolidation (excluding cases with infiltrates only) and found a population attributable fraction of 42% for *S. pneumoniae*.[3] This value is consistent with reductions in radiological pneumonia hospitalisations following the introduction of PCV in many countries.[25-28]

Our observation of co-infection with two (21%), three (8%), and four or more pathogens (6%) underscores the polymicrobial nature of lobar pneumonia. Bacterial-bacterial and bacterial-viral co-infections were of similar prevalence. In the setting of co-infection, the estimation of aetiological proportions due to individual pathogens remains a challenge with all aetiological pathogens necessarily contributing to more than 100% of cases. The importance of co-infections, temporal pathogenesis, and the interplay of viral upper and bacterial lower respiratory infections, raises the potential for vaccine interventions to impact pathogenesis involving non-target pathogens. The synergistic role of *S. pneumoniae* has already been demonstrated in a vaccine probe study showing the administration of PCV prevented hospitalization with viral-associated lower respiratory disease.[29]

The correlation we observed between *M. catarrhalis* and *S. pneumoniae* is intriguing. This may be explained by true synergism or by correlation alone given these organisms commonly co-colonize the upper respiratory tract. Aspiration of upper respiratory flora in the pathogenesis of lobar



For peer review only - http://bmjopen.bmj.com/site/about/guidelines.xhtml

For peer review only

pneumonia would result in co-detection of such bacteria in lung tissue, if bacteria were able to avoid neutrophil killing and other clearance mechanisms.

We estimated the effectiveness of PCV against non-bacteraemic pneumococcal pneumonia, which has not been possible in most trials. Among adults in the Netherlands the efficacy of one dose of PCV13 was 45% to prevent non-invasive vaccine-type pneumococcal pneumonia and 75% to prevent vaccine-type invasive disease. Our estimates of PCV effectiveness against vaccine-type (OR 0.17; 95% CI 0.06, 0.51) and all pneumococcal pneumonia (OR 0.42; 95% CI 0.16, 1.05) are similar to the Gambian PCV9 trial estimates of efficacy against lung aspirate positive vaccine-type (73%) and all pneumococcal pneumonia (68%).[30]

The main strength of our study is the inclusion of a significant number of lung aspirate specimens combined with a sensitive and specific multiplex PCR assay. Our study was limited by aetiological testing of only 160/741 patients with lobar pneumonia, the limited range of potential pathogens detected and the limited sample size. The multiplex assay excluded measles and *M. tuberculosis*. The PERCH study found no cases of *M. tuberculosis* in lung or pleural aspirates but it was isolated in The Gambia in 7/255 induced sputum specimens.[2] The already cited Gambian study of 53 lung specimens found no cases of *M. tuberculosis.*[9] Our analyses excluded *Legionella* and *Klebsiella* sp. due to poor assay specificity. We were unable to detect the aetiological pathogen(s) in 28% of patients with a lung aspirate. Our cross-sectional design was not able to investigate the temporal aspects of pneumonia pathogenesis. The limited duration of our study may also introduce potential bias due to variation in the seasonal transmission of individual pathogens.

Understanding the contribution of less prevalent pathogens in lobar pneumonia, the age distribution of pathogen aetiology, and questions concerning co-infection and synergism will require larger sample sizes. More sensitive and specific multiplex assays may identify additional pathogens. Studies



For peer review only - http://bmjopen.bmj.com/site/about/guidelines.xhtml

of pneumonia aetiology, and childhood pneumonia in general, should carefully consider the use of specific case definitions, for example separating bronchiolitis and lobar pneumonia phenotypes, to avoid heterogeneity in outcome measurements.[31] Longitudinal studies of pneumonia pathogenesis, or vaccine probe studies (such as with an RSV vaccine), may help determine the relationships between viruses and bacteria. Studies of pathogen gene expression in the lung[32] may reveal new therapeutic approaches.

Our study provides important information concerning the aetiology of lobar pneumonia in a setting with significant child mortality during the period of introduction of PCV. Our findings may not be generalizable to settings with different levels of vaccine coverage and nasopharyngeal bacterial carriage. Further studies using lung aspirates will address a number of remaining important questions.

For peer review only



For peer review only - http://bmjopen.bmj.com/site/about/guidelines.xhtml

**Acknowledgments**

We are grateful to the staff at Basse District Hospital and Bansang Hospital and staff of the Pneumococcal Surveillance Project who provided clinical evaluation and care for the patients. The Regional Health Teams in Upper and Central River Region provided logistic support.

**Contributors**

GM conceived and designed the study, conducted the analysis, and wrote the first draft of the manuscript. JM and EM conducted multiplex qPCR analyses and reviewed the manuscript. MN, JP, AF, BA, and IH enrolled the patients, collected the specimens and reviewed the manuscript. AM conducted conventional microbiological analyses and reviewed the manuscript. BG and PH advised on analysis and reviewed the manuscript. All authors approved the final version of the manuscript for submission.

**Funding**

This work was supported by the Bill & Melinda Gates Foundation [grant number OPP1020327] and the Medical Research Council Unit The Gambia at London School of Hygiene & Tropical Medicine.

**Competing interests**

The authors declare no competing interests.

**Patient consent for publication**

Not required.

**Ethics approval**

Ethical approval was granted for the study by the Gambia Government/Medical Research Council (UK) Joint Ethics Committee (numbers 1087 and 1247). Written informed consent was obtained from patients or guardian for all study procedures.

**Provenance and peer review**

No part of this work has been written by a medical writer. Some of the findings of this study were presented at the 66$^{th}$ annual meeting of the American Society of Tropical Medicine & Hygiene

For peer review only

(abstract #: 17-A-1389). A copy of the originally submitted manuscript was uploaded to the medRxiv preprint website; https://medrxiv.org/cgi/content/short/2021.07.02.21259855v1.

**Data availability statement**

Data are available upon reasonable request to the MRCG Scientific Coordinating Committee and Gambia Government/MRCG Joint Ethics Committee. Deidentified patient data may be requested from the MRCG Data Management and Archives department.

**ORCID ID**

Grant Mackenzie 0000-0002-4994-2627

Jessica McLellan 0000-0002-5486-9620

Eunice Machuka 0000-0002-1142-3442

Malick Ndiaye not available

Jayani Pathirana 0000-0001-9678-4759

Augustin Fombah not available

Baderinwa Abatan not available

Ilias Hossain 0000-0002-0571-4702

Ahmed Manjang 0000-0003-4475-3090

Brian Greenwood 0000-0001-5725-9118

Philip C Hill 0000-0002-7006-0549

**Exclusive licence**

I, the Submitting Author have the right to grant and do grant on behalf of all authors of the Work (as defined in the below author licence), an exclusive licence and/or a non-exclusive licence for contributions from authors who are: i) UK Crown employees; ii) where BMJ has agreed a CC-BY licence shall apply, and/or iii) in accordance with the terms applicable for US Federal Government officers or employees acting as part of their official duties; on a worldwide, perpetual, irrevocable, royalty-free basis to BMJ Publishing Group Ltd ("BMJ") its licensees and where the relevant Journal is

For peer review only

co-owned by BMJ to the co-owners of the Journal, to publish the Work in any BMJ product and to exploit all rights, as set out in our licence.

**Figure legends**

**Figure 1. Study profile**

**References**

(1) Selwyn BJ. The epidemiology of acute respiratory tract infection in young children: comparison of findings from several developing countries. Coordinated Data Group of BOSTID Researchers. *Rev Infect Dis* 1990;12:S870-88.

(2) PERCH Study Group. Causes of severe pneumonia requiring hospital admission in children without HIV infection from Africa and Asia: the PERCH multi-country case-control study. *Lancet* 2019;394:757-79.

(3) Benet T, Sanchez P, V, Messaoudi M, *et al*. Microorganisms Associated With Pneumonia in Children <5 Years of Age in Developing and Emerging Countries: The GABRIEL Pneumonia Multicenter, Prospective, Case-Control Study. *Clin Infect Dis* 2017;65:604-12.

(4) Zar HJ, Barnett W, Stadler A, Gardner-Lubbe S, Myer L, Nicol MP. Aetiology of childhood pneumonia in a well vaccinated South African birth cohort: a nested case-control study of the Drakenstein Child Health Study. *Lancet Respir Med* 2016;4:463-72.

(5) Shann F, Gratten M, Germer S, Linnemann V, Hazlett D, Payne R. Aetiology of pneumonia in children in Goroka Hospital, Papua New Guinea. *Lancet* 1984;2:537-41.

(6) Kalra SK, Sasidharan T, Vatwani V, Sarkar P. Lung puncture: a diagnostic aid in childhood pneumonia. *Indian Pediatr* 1981;18:727-30.

(7) Wall RA, Corrah PT, Mabey DC, Greenwood BM. The etiology of lobar pneumonia in the Gambia. *Bull World Health Organ* 1986;64:553-8.

(8) Silverman M, Stratton D, Diallo A, Egler LJ. Diagnosis of acute bacterial pneumonia in Nigerian children. Value of needle aspiration of lung. *Arch Dis Child* 1977;52:925-31.

(9) Howie SR, Morris GA, Tokarz R, *et al*. Etiology of severe childhood pneumonia in the Gambia, West Africa, determined by conventional and molecular microbiological analyses of lung and pleural aspirate samples. *Clin Infect Dis* 2014;59:682-5.

(10) Duke T. What the PERCH study means for future pneumonia strategies. *Lancet* 2019;394:714-6.

(11) Mackenzie GA, Plumb ID, Sambou S, *et al*. Monitoring the introduction of pneumococcal conjugate vaccines into West Africa: design and implementation of a population-based surveillance system. *PLoS Med* 2012;9:e1001161.



For peer review only - http://bmjopen.bmj.com/site/about/guidelines.xhtml

(12) Mackenzie GA, Hill PC, Jeffries DJ, *et al*. Effect of the introduction of pneumococcal conjugate vaccination on invasive pneumococcal disease in The Gambia: a population-based surveillance study. *Lancet Infect Dis* 2016;16:703-11.

(13) Mackenzie GA, Hill PC, Sahito SM, *et al*. Impact of the introduction of pneumococcal conjugate vaccination on pneumonia in The Gambia: population-based surveillance and case-control studies. *Lancet Infect Dis* 2017;17:965-73.

(14) National AIDS Control Programme. Republic of The Gambia, HIV Sentinel Surveillance Report 2011. Banjul: Ministry of Health & Social Welfare; 2012.

(15) World Health Organization Pneumonia Vaccine Trial Investigators' Group. Standardization of interpretation of chest radiographs for the diagnosis of pneumonia in children. World Health Organization, Department of Vaccines and Biologicals. 2001. Available at: http://www.who.int/vaccines-documents. Accessed 5 October 2004.

(16) Ideh RC, Howie SR, Ebruke B, *et al*. Transthoracic lung aspiration for the aetiological diagnosis of pneumonia: 25 years of experience from The Gambia. *Int J Tuberc Lung Dis* 2011;15:729-35.

(17) Adegbola RA, Falade AG, Sam BE, *et al*. The etiology of pneumonia in malnourished and well-nourished Gambian children. *Pediatr Infect Dis J* 1994;13:975-82.

(18) O'Neill KP, Lloyd-Evans N, Campbell H, Forgie IM, Sabally S, Greenwood BM. Latex agglutination test for diagnosing pneumococcal pneumonia in children in developing countries. *BMJ* 1989;298:1061-4.

(19) Driscoll AJ, Karron RA, Morpeth SC, *et al*. Standardization of Laboratory Methods for the PERCH Study. *Clin Infect Dis* 2017;64:S245-S252.

(20) Mackenzie GA, Hill PC, Jeffries DJ, *et al*. Impact of the introduction of pneumococcal conjugate vaccination on invasive pneumococcal disease and pneumonia in The Gambia: 10 years of population-based surveillance. *Lancet Infect Dis* 2021 [in press].

(21) Zaman SM, Howie SR, Ochoge M, *et al*. Impact of routine vaccination against Haemophilus influenzae type b in The Gambia: 20 years after its introduction. *J Glob Health* 2020;10:010416.

(22) Standard Treatment Guidelines.The Gambia: Ministry of Health; 2017.

(23) Scott JAG, Hall AJ. The value and complications of percutaneous transthoracic lung aspiration for the etiologic diagnosis of community-acquired pneumonia. *Chest* 1999;116:1716-32.

(24) Diakparomre MA, Obi JO. Aetiological diagnosis of pneumonia in childhood by lung puncture. *Nigerian J of Paediatrics* 1981;8:61-4.

(25) Greenberg D, Givon-Lavi N, Ben-Shimol S, Ziv JB, Dagan R. Impact of PCV7/PCV13 introduction on community-acquired alveolar pneumonia in children <5 years. *Vaccine* 2015;33:4623-9.


For peer review only



For peer review only - http://bmjopen.bmj.com/site/about/guidelines.xhtml

(26) Pirez MC, Algorta G, Cedres A, *et al*. Impact of universal pneumococcal vaccination on hospitalizations for pneumonia and meningitis in children in Montevideo, Uruguay. *Pediatr Infect Dis J* 2011;30:669-74.

(27) Hammitt LL, Etyang AO, Morpeth SC, *et al.* Effect of ten-valent pneumococcal conugate vaccine on invasive pneumococcal disease and nasopharyngeal carriage in Kenya: a longitudinal surveillance study. *Lancet* 2019;393:2146-54.

(28) Mackenzie GA, Hill PC, Jeffries DJ, *et al*. Impact of the introduction of pneumococcal conjugate vaccination on invasive pneumococcal disease and pneumonia in The Gambia: 10 years of population-based surveillance. *Lancet Infect Dis* 2021;21:1293-302.

(29) Madhi SA, Klugman KP. A role for Streptococcus pneumoniae in virus-associated pneumonia. *Nat Med* 2004;10:811-3.

(30) Cutts FT, Zaman SM, Enwere G, *et al*. Efficacy of nine-valent pneumococcal conjugate vaccine against pneumonia and invasive pneumococcal disease in The Gambia: randomised, double-blind, placebo-controlled trial. *Lancet* 2005;365:1139-46.

(31) Mackenzie G. The definition and classification of pneumonia. *Pneumonia* 2016;8:14.

(32) Dunne EM, Hua Y, Salaudeen R, *et al*. Insights into pneumococcal pneumonia using lung aspirates and nasopharyngeal swabs collected from pneumonia patients in The Gambia. *J Infect Dis* **2020**; doi 10.1093/infdis/jiaa186.



For peer review only - http://bmjopen.bmj.com/site/about/guidelines.xhtml

**Figure 1. Study profile**

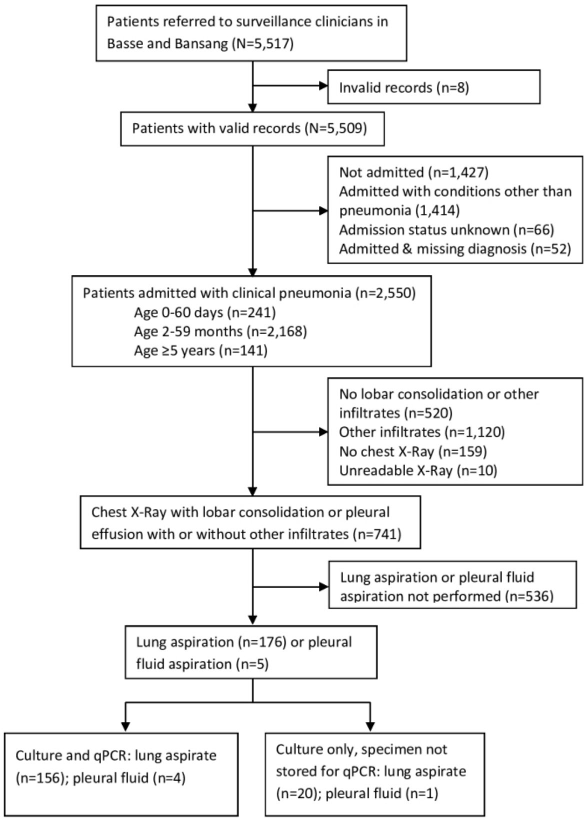


210x297mm (200 x 200 DPI)

# Aetiology of Lobar Pneumonia Determined by Multiplex Molecular Analyses of Lung and Pleural Aspirate Samples in The Gambia: Findings from Population-based Pneumonia Surveillance

### *Methods for multiplex PCR assay*

The laboratory, staff, and methods used to analyse the specimens were the same to those used in the PERCH study which was also conducted in The Gambia at a similar time. Total nucleic acid was extracted from a 200µl aliquot of lung and pleural aspirates (easyMAG, bioMériux, France) with an internal control. Extracts were subjected to quantitative multiplex PCR (Fast-track Diagnostics Resp-33 kit, Sliema, Malta) for a panel of 33 respiratory bacteria, fungi, and viruses with internal positive and negative control. The assay was structured in eight component multiplex sub-assays with three or four targets run on one plate. We used a Bio-Rad CFX96 thermocycler with programming as recommended by the manufacturer. Standard PCR curves were derived from plasmid standards during the testing to calculate pathogen load from cycle threshold values. The multiplex PCR included the following targets:

- *S. pneumoniae* (lytA),
- *Haemophilus influenze* sp. (ompP6),
- *H. influenzae* type b (bexA),
- *S. aureus* (shkv),
- *Chlamydia pneumoniae* (RNApbc),
- *Moraxella catarrhalis* (copB),
- *Klebsiella pneumoniae* (khe),
- *Legionella* sp. (16SrRNA),
- *Pneumocystis jirovecii* (mtlsurRNA),
- *Bordetella pertussis* (is481),
- *Salmonella* sp. (ttrB),
- Influenza A (pos1), B (seg8ns1nep) and C (mtx),
- Cytomegalovirus (us7&8),
- Parainfluenza virus 1 (hnmRNA), 2 (hnmRNA), 3 (hnmRNA) and 4 (fus),
- Rhinovirus (utr),
- Coronaviruses NL63 (ncpn), 229E (ncpn), OC43 (ncpn) and HKU1 (ncpn),
- Respiratory syncytial virus A (nucap) and B (numRNA),
- Metapneumovirus A (fuglF) and B (fuglF),
- Adenovirus (hex),
- Bocavirus (np1),
- Enterovirus (dom4&5),
- Parechovirus (utr),
- *Mycoplasma pneumoniae* (adP1),



For peer review only - http://bmjopen.bmj.com/site/about/guidelines.xhtml

Data were not used for *K. pneumoniae* and Legionella spp. Interpretation for some targets required combinations of results: if rhinovirus only was detected then the specimen was deemed rhinovirus positive, whereas if rhinovirus and enterovirus were detected then the specimen was deemed enterovirus positive; if *H. influenzae* type b and *H. influenzae* were detected the specimen was deemed positive for *H. influenzae* type b, whereas if *H. influenzae* only was detected the specimen was deemed positive for *H. influenzae* non-type b.

***Clinical characteristics of patients***

Table 1 in the manuscript describes the characteristics of the patients in three categories: no lobar consolidation, lobar consolidation and no lung/pleural aspirate, and lobar consolidation and lung/pleural aspirate. Compared to patients without lobar pneumonia, those with lobar pneumonia had greater respiratory rate ($p$<0.0001), lower oxygen saturation ($p$=0.034), and less wheeze ($p$<0.0001), whereas heart rate ($p$=0.59), temperature ($p$=0.73), prostration ($p$=0.25), weight-for-height z-score <-3 in young children ($p$=0.28) and severe underweight in older children and adults ($p$=0.36) were not significantly different. Respiratory rate ($p$=0.50), heart rate ($p$=0.20), temperature ($p$=0.12), prostration ($p$=0.20), weight-for-height z-score <-3 in young children ($p$=0.62), and severe underweight in older children and adults ($p$=0.86) were not significantly different in patients with lobar pneumonia who did or did not have a lung aspirate, although wheeze was more frequent in patients without lung aspirate (76/562 versus 11/181, $p$=0.007) and oxygen saturation was greater ($p$=0.017). Bacteremia was more likely in patients who had a lung aspirate (31/178, 17%) compared to those without a lung aspirate, irrespective of whether lobar pneumonia was present on chest radiograph (113/2119, 5%). Ninety-six patients died (3.8%) with similar proportions in the three clinical categories.



For peer review only - http://bmjopen.bmj.com/site/about/guidelines.xhtml

***Quantification of pathogen load***

The greatest pathogen load in lung specimens was associated with *S. pneumoniae* (median 5.34 [IQR 3.73, 6.24] $log_{10}$ copies/ml), *H. influenzae* non-type b (median 6.07 [IQR 5.32, 6.86] $log_{10}$ copies/ml) and parainfluenza virus (PIV) 1 (median 6.46 [IQR 4.74, 10.93] $log_{10}$ copies/ml) positive specimens (Supplementary Table 1). Low pathogen load was associated with *S. aureus* (median 2.15 [IQR 1.68, 4.14] $log_{10}$ copies/ml), bocavirus (median 2.77 [IQR 2.19, 3.40] $log_{10}$ copies/ml]), and cytomegalovirus (2.57 [IQR 2.38, 3.71] $log_{10}$ copies/ml) positive specimens.

**Supplementary table 1. Organism-specific quantification of pathogen load in 156 lung and 4 pleural aspirate specimens**

| Organism | Quantification of organism (median[IQR]; min, max); $log_{10}$ copies per ml |
|---|---|
| **Bacteria** | |
| *Streptococcus pneumoniae* (n=68) | 5.34 (3.73 – 6.24); 1.44, 9.58 |
| *Staphylococcus aureus* (n=26) | 2.15 (1.68 – 4.14); 1.43, 8.49 |
| *Haemophilus influenzae* type b (n=11) | 4.18 (2.26 – 6.30); 1.56, 9.11 |
| *Moraxella catarrhalis* (n=8) | 4.40 (3.71 – 5.50); 2.63, 6.30 |
| *Salmonella* species (n=8) | 3.01 (1.74 – 5.29); 0.86, 9.07 |
| *Haemophilus influenzae* non-type b (n=6) | 6.07 (5.32 – 6.86); 4.88, 8.21 |
| *Bordetella pertussis* (n=4) | undef (undef); 0.30, 4.32 |
| *Chlamydia pneumonia* (n=3) | 3.60 (undef); 2.13, 4.73 |
| **Viruses** | |
| Bocavirus (n=11) | 2.77 (2.19 – 3.40); 1.53, 4.76 |
| Parainfluenza 1 (n=8) | 6.46 (4.74 – 10.93); 4.32, 12.50 |
| Influenza C (n=7) | 4.47 (4.21 – 5.64); 3.72, 6.85 |
| Cytomegalovirus (n=6) | 2.57 (2.38 – 3.71); 1.45, 5.89 |
| Coronavirus HKU1 (n=4) | 3.93 (undef); 3.77, 4.46 |
| Coronavirus 43 (n=4) | 4.77 (undef); 4.25, 5.37 |
| Respiratory syncytial virus (n=3) | 6.59 (undef); 5.07, 7.07 |
| **Fungi** | |
| *Pneumocystis jirovecii* (n=9) | 2.82 (2.52 – 3.37); 2.14, 7.42 |

Note: organisms listed were detected in three or more of 160 specimens. *B. pertussis* PCR Ct values were too great to allow quantification for three of seven specimens. The results of pathogen quantification in lobar pneumonia are subject to variation in the small volumes of specimen obtained and its dilution in 1ml of sterile saline.

For peer review only

*Effectiveness of PCV to prevent pneumococcal pneumonia*

**Supplementary table 2. Association of pneumococcal pneumonia with PCV vaccination status**

| **Pneumonia aetiology by culture of blood or lung/pleural aspirate** | **Number of PCV doses (PCV7 or PCV13)** | | **Total N** | **Odds ratio (95% CI)** |
|---|---|---|---|---|
| | ≥2 doses | 0 doses | | |
| Age 2-11 months | N=540 | N=184 | | |
| Culture pneumococcal | 5 | 5 | 10 | |
| Culture non-pneumococcal | 535 | 179 | 714 | 0.33 (0.08, 1.47) |
| Proportion culture pneumococcal | 0.009 | 0.027 | 724 | |
| Age 12-23 months | N=515 | N=81 | | |
| Culture pneumococcal | 15 | 2 | 17 | |
| Culture non-pneumococcal | 500 | 79 | 560 | 1.19 (0.27, 10.9) |
| Proportion culture pneumococcal | 0.029 | 0.025 | 577 | |
| Age 2-4 years | N=230 | N=218 | | |
| Culture pneumococcal | 9 | 15 | 24 | |
| Culture non-pneumococcal | 221 | 203 | 424 | 0.55 (0.21, 1.38) |
| Proportion culture pneumococcal | 0.039 | 0.069 | 448 | |
| Combined age strata 2-59 months, [a]M-H age-stratified odds ratio = 0.57 (0.31, 1.06), [b]*p*=0.076 | | | | |
| **Pneumonia aetiology by culture of blood or lung/pleural aspirate or PCR on lung/pleural aspirate** | | | | |
| Age 2-11 months | N=540 | N=184 | | |
| PCR or culture pneumococcal | 8 | 8 | 16 | |
| Not PCR or culture pneumococcal | 532 | 176 | 684 | 0.33 (0.11, 1.03) |
| Proportion PCR or culture pneumococcal | 0.015 | 0.043 | 708 | |
| Age 12-23 months | N=515 | N=81 | | |
| PCR or culture pneumococcal | 22 | 4 | 26 | |
| Not PCR or culture pneumococcal | 493 | 77 | 570 | 0.86 (0.28, 3.52) |
| Proportion PCR or culture pneumococcal | 0.043 | 0.049 | 596 | |
| Age 2-4 years | N=230 | N=218 | | |
| PCR or culture pneumococcal | 13 | 21 | 34 | |
| Not PCR or culture pneumococcal | 217 | 197 | 414 | 0.56 (0.25, 1.21) |
| Proportion PCR or culture pneumococcal | 0.057 | 0.096 | 448 | |
| Combined age strata 2-59 months, [a]M-H age-stratified odds ratio = 0.54 (0.33, 0.90), [b]*p*=0.017 | | | | |

[a]Mantel-Haenzel age-stratified odds ratio. [b]Fisher's exact *p*-value.



For peer review only

STROBE Statement—Checklist of items that should be included in reports of ***cross-sectional studies***

| | Item No | Recommendation | Page No |
|---|---|---|---|
| **Title and abstract** | 1 | (*a*) Indicate the study's design with a commonly used term in the title or the abstract | 1 |
| | | (*b*) Provide in the abstract an informative and balanced summary of what was done and what was found | 2 |
| **Introduction** | | | |
| Background/rationale | 2 | Explain the scientific background and rationale for the investigation being reported | 4 |
| Objectives | 3 | State specific objectives, including any prespecified hypotheses | 4 |
| **Methods** | | | |
| Study design | 4 | Present key elements of study design early in the paper | 5 |
| Setting | 5 | Describe the setting, locations, and relevant dates, including periods of recruitment, exposure, follow-up, and data collection | 5 |
| Participants | 6 | (*a*) Give the eligibility criteria, and the sources and methods of selection of participants | 5 |
| Variables | 7 | Clearly define all outcomes, exposures, predictors, potential confounders, and effect modifiers. Give diagnostic criteria, if applicable | 7 |
| Data sources/ measurement | 8* | For each variable of interest, give sources of data and details of methods of assessment (measurement). Describe comparability of assessment methods if there is more than one group | 6 |
| Bias | 9 | Describe any efforts to address potential sources of bias | 7 |
| Study size | 10 | Explain how the study size was arrived at | 8 |
| Quantitative variables | 11 | Explain how quantitative variables were handled in the analyses. If applicable, describe which groupings were chosen and why | 7 |
| Statistical methods | 12 | (*a*) Describe all statistical methods, including those used to control for confounding | 7 |
| | | (*b*) Describe any methods used to examine subgroups and interactions | 7 |
| | | (*c*) Explain how missing data were addressed | nd |
| | | (*d*) If applicable, describe analytical methods taking account of sampling strategy | 7 |
| | | (*e*) Describe any sensitivity analyses | nd |
| **Results** | | | |
| Participants | 13* | (a) Report numbers of individuals at each stage of study—eg numbers potentially eligible, examined for eligibility, confirmed eligible, included in the study, completing follow-up, and analysed | 8 |
| | | (b) Give reasons for non-participation at each stage | 8 |
| | | (c) Consider use of a flow diagram | 8 |
| Descriptive data | 14* | (a) Give characteristics of study participants (eg demographic, clinical, social) and information on exposures and potential confounders | 8 |
| | | (b) Indicate number of participants with missing data for each variable of interest | 9 |
| Outcome data | 15* | Report numbers of outcome events or summary measures | 9 |

For peer review only

| | | | |
|---|---|---|---|
| Main results | 16 | (*a*) Give unadjusted estimates and, if applicable, confounder-adjusted estimates and their precision (eg, 95% confidence interval). Make clear which confounders were adjusted for and why they were included | 11-13 |
| | | (*b*) Report category boundaries when continuous variables were categorized | na |
| | | (*c*) If relevant, consider translating estimates of relative risk into absolute risk for a meaningful time period | na |
| Other analyses | 17 | Report other analyses done—eg analyses of subgroups and interactions, and sensitivity analyses | 10 |
| **Discussion** | | | |
| Key results | 18 | Summarise key results with reference to study objectives | 15 |
| Limitations | 19 | Discuss limitations of the study, taking into account sources of potential bias or imprecision. Discuss both direction and magnitude of any potential bias | 17 |
| Interpretation | 20 | Give a cautious overall interpretation of results considering objectives, limitations, multiplicity of analyses, results from similar studies, and other relevant evidence | 18 |
| Generalisability | 21 | Discuss the generalisability (external validity) of the study results | 18 |
| **Other information** | | | |
| Funding | 22 | Give the source of funding and the role of the funders for the present study and, if applicable, for the original study on which the present article is based | 19 |

*Give information separately for exposed and unexposed groups.

**Note:** An Explanation and Elaboration article discusses each checklist item and gives methodological background and published examples of transparent reporting. The STROBE checklist is best used in conjunction with this article (freely available on the Web sites of PLoS Medicine at http://www.plosmedicine.org/, Annals of Internal Medicine at http://www.annals.org/, and Epidemiology at http://www.epidem.com/). Information on the STROBE Initiative is available at www.strobe-statement.org.

For peer review only
